# Emerging Separation Applications of Surface Superwettability

**DOI:** 10.3390/nano12040688

**Published:** 2022-02-18

**Authors:** Jiale Yong, Qing Yang, Xun Hou, Feng Chen

**Affiliations:** 1 State Key Laboratory for Manufacturing System Engineering and Shaanxi Key Laboratory of Photonics Technology for Information, School of Electronic Science and Engineering, Xi’an Jiaotong University, Xi’an 710049, China; jlyong@xjtu.edu.cn (J.Y.); houxun@mail.xjtu.edu.cn (X.H.); 2 School of Mechanical Engineering, Xi’an Jiaotong University, Xi’an 710049, China; yangqing@mail.xjtu.edu.cn

**Keywords:** solid/liquid/gas separation, oil/water separation, water/gas separation, superwettability, superhydrophobicity

## Abstract

Human beings are facing severe global environmental problems and sustainable development problems. Effective separation technology plays an essential role in solving these challenges. In the past decades, superwettability (e.g., superhydrophobicity and underwater superoleophobicity) has succeeded in achieving oil/water separation. The mixture of oil and water is just the tip of the iceberg of the mixtures that need to be separated, so the wettability-based separation strategy should be extended to treat other kinds of liquid/liquid or liquid/gas mixtures. This review aims at generalizing the approach of the well-developed oil/water separation to separate various multiphase mixtures based on the surface superwettability. Superhydrophobic and even superoleophobic surface microstructures have liquid-repellent properties, making different liquids keep away from them. Inspired by the process of oil/water separation, liquid polymers can be separated from water by using underwater superpolymphobic materials. Meanwhile, the underwater superaerophobic and superaerophilic porous materials are successfully used to collect or remove gas bubbles in a liquid, thus achieving liquid/gas separation. We believe that the diversified wettability-based separation methods can be potentially applied in industrial manufacture, energy use, environmental protection, agricultural production, and so on.

## 1. Introduction

Human beings are facing severe global environmental problems and sustainable development problems. At present, the main environmental issues that have aroused international concern include global climate change, acid rain pollution, freshwater resource crisis, energy shortage, a sharp decrease in biodiversity, toxic chemical pollution, air pollution, marine pollution, and so on [1,2,3,4,5,6]. For example, with the continuous growth of global energy demand, oil leakage accidents frequently occur, and a large volume of industrial oily wastewater is discharged, leading to serious ecological and environmental problems [7,8,9,10]. Effective separation technology plays an essential role in solving these problems. Solid, liquid, and gas phases are common forms of matter. They are usually interwoven with each other to form different kinds of mixtures. The separations of gas, liquid, and solid multiphase mixtures are of great significance [11,12,13,14,15]. Various separation materials and methods have been developed to separate the multiphase mixtures [16,17,18,19,20,21,22,23,24,25]. As a fundamental physicochemical property, the surface wettability of a solid substrate is a visible result of the interaction between different phases at the solid-liquid-gas interface [26,27,28,29,30,31,32,33,34,35]. For the extrema state, superwettability enables the materials to have a significant ability to absorb or repel liquids [36,37,38,39,40]. The lotus leaf with superhydrophobicity is difficult to be wetted by water [41,42,43,44]. Water droplets can easily roll away on the lotus leaf [45,46,47]. Fish scales have underwater superoleophobicity and superaerophobicity and are able to repel oils and bubbles in water, preventing fish skin from being contaminated [48,49]. Recently, extreme surface wettability has had great success in achieving oil/water separation [50,51,52,53,54,55,56,57,58,59,60]. Feng et al. first used a superhydrophobic and superoleophilic mesh to separate the mixture of water and oil [61]. Water was repelled by the mesh and thus maintained above the mesh, while oil wetted the mesh and passed through the mesh. Xue et al. further developed a method to separate oil/water mixtures by using a superhydrophilic and underwater superoleophobic mesh that allowed water to pass through but intercepted oils [62]. It is widely demonstrated that the mixture of oils and water can be effectively separated by porous materials with either superhydrophobicity or underwater superoleophobicity [63,64,65,66,67,68,69,70]. Such a separation is supported by the phenomenon that oils and water have different wetting behaviors on those superwetting separation materials [71,72,73,74,75,76,77,78,79,80]. The oil/water mixture is just the tip of the iceberg, and many other mixtures also need to be separated. There is still a significant challenge: can the superwettability-based separation method be extended to separate different mixtures besides the great success in oil/water separation? The application scope of the superwettability-based separation should be significantly enlarged, and the generalization of such a separation strategy is of great significance.

**Figure 1:**
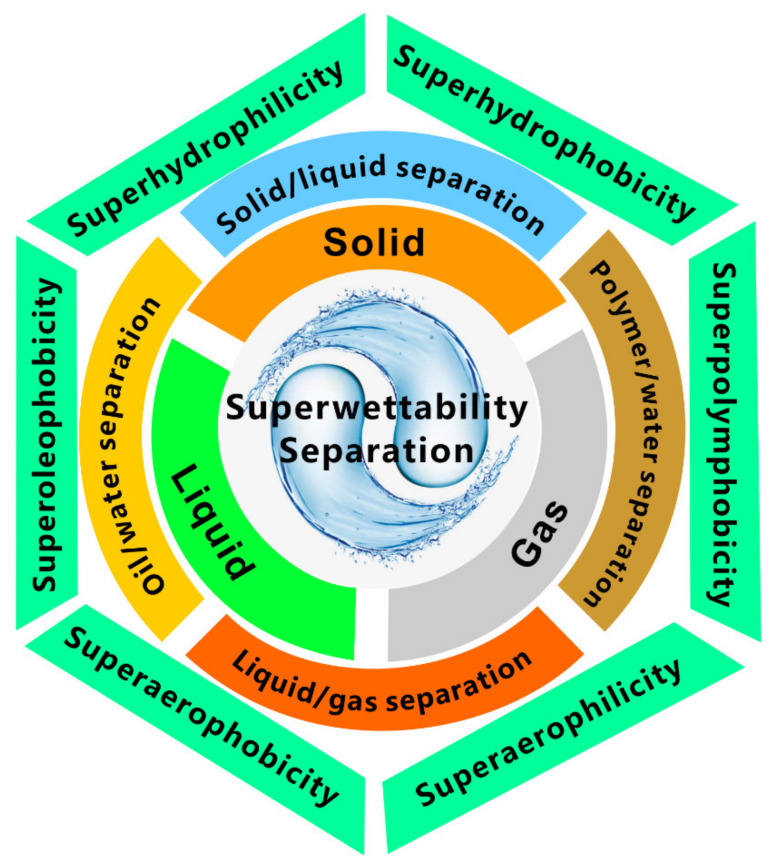
Solid/liquid/gas separation based on the surface superwettability.

This review will summarize the current progress of the novel separation applications of superwetting materials (Figure 1). We aim at promoting the well-developed oil/water separation process based on the superwettability for the treatment of other types of liquid/liquid or liquid/gas mixtures, such as separating liquid polymer and gas from water. Classical wetting models are first introduced as the background, which is the basis for achieving various superwetting properties (Section 2). The next part focuses on the function of superhydrophobicity and superoleophobicity in separating liquids from solid materials (Section 3). Inspired by nature, a large number of materials with strong liquid repellence have been fabricated, such as superhydrophobic materials and superoleophobic materials. The superwettability allows the materials to repel liquids and thus makes liquid away from a solid substrate. Then, the successful application of the porous materials with superwettability in oil/water separation is introduced (Section 4). Three typical types of oil/water separation materials are taken as examples, including superhydrophobic porous membrane, underwater superoleophobic porous membrane, and superhydrophobic 3D oil-absorption materials. Inspired by the process of oil/water separation, the separation of the mixture of water and liquid polymers and the mixture of gas bubbles and water can also be achieved by the superwetting materials. Usually, liquid polymers readily adhere to a solid surface and are difficult to remove because they have a higher viscosity, lower fluidity, and more complex composition than pure water and oils. The next part shows using an underwater superpolymphobic porous membrane to separate liquid polymers from water (Section 5). After that, we present the strategy of removing or collecting tiny gas bubbles in water by the underwater superaerophobic and superaerophilic porous materials (Section 6). Finally, a discussion of current challenges and prospects of the diversified wettability-based separation processes are briefly discussed (Section 7).

## 2. Theoretical Basis about Wettability (Typical Wetting States)

**Figure 2:**
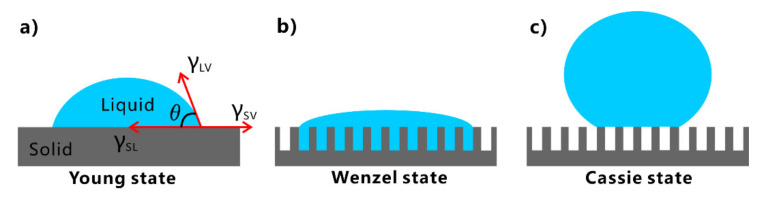
Typical contact states between a liquid droplet and solid surface: (**a**) Young contact state, (**b**) Wenzel contact state, and (**c**) Cassie contact state.

Wettability is an important property of solid materials. When a liquid droplet comes in contact with a solid surface in the air, a three-phase contact line is formed at the junction of the solid, liquid, and gas phases. The contact line continues to expand outward until the droplet reaches a static state. For a droplet on the solid surface, the angle formed between the solid-liquid contact surface and the tangent line of the droplet at the three-phase line is called contact angle (CA, *θ* is used in the equation), as shown in Figure 2a [26,36]. When the surface is gradually tilted until the droplet can roll away or slide down, the tilted angle is called the sliding angle (SA). Surface wettability can be investigated by the CA and SA measurements [26,36,81]. The CA value can reflect the static wetting characteristics of the solid surface. With water as an example, the surface is generally considered to be hydrophilic when *θ* < 90°, while the surface is hydrophobic when *θ* > 90°. For the extreme case, the surface is regarded as superhydrophilic at *θ* < 10° and superhydrophobic at *θ* > 150° [26,82]. On the other hand, dynamic wettability is usually investigated by a SA. The surface with a large SA value has high adhesion to liquid. In contrast, the surface with low SA has very low adhesion to liquid.

The wettability of a liquid droplet on a perfectly smooth solid surface can be described by the Young equation (Figure 2a) [82,83]:

(1) $$\cos\theta =\frac{\gamma_{SV}-\gamma_{SL}}{\gamma_{\mathrm{LV}}}$$

where *θ* is the CA of the liquid droplet. $\gamma_{\mathrm{SL}}$, $\gamma_{\mathrm{SV}}$, and $\gamma_{\mathrm{LV}}$ are the solid-liquid, solid-vapor, and liquid-vapor interfacial energies, respectively.

For an actual solid surface that usually has rough morphology, Wenzel introduced the factor of surface roughness, *r*, into Young’s equation after considering the influence of rough surface structure on the wettability [84]. The *r* is defined as the ratio of the actual surface area to the projected surface area of the uneven surface. In this case, the liquid fills the microstructure of the solid surface (Figure 2b). For a liquid droplet in the Wenzel state, the measured/apparent CA (θ*) can be expressed by the Wenzel equation [36,85]:

(2) $$\cos\theta^{*}=r\frac{\gamma_{SL}-\gamma_{SV}}{\gamma_{\mathrm{LV}}}=r\cos\theta$$

where *θ* is the Young’s CA of a liquid droplet on the flat solid surface. The surface roughness can enhance the inherent wettability of the solid surface either for an intrinsically hydrophobic or hydrophilic substrate [36,82].

In some cases, the liquid cannot penetrate the microstructure of a rough surface [83]. There is a layer of trapped air between the solid surface and the liquid. The contact interface between liquid and the solid surface consists of liquid-solid contact and liquid-air contact (Figure 2c). This wettability situation can be given by the Cassie equation [36,86]:

(3) $$\cos\theta^{*}=f_{1}\cos\theta_{1}+f_{2}\cos\theta_{2}$$

where θ* is the measured CA of a liquid droplet on such a composite surface. *θ*_1_ and *θ*_2_ are the CA of the liquid droplet on the solid phase and air phase, respectively. *f_1_* and *f_2_* are the area ratio of liquid-solid contact and the liquid-air contact, respectively. Liquid droplet is perfectly spherical in the air so that its CA on the gas surface can be considered as 180°. For a solid surface with microstructure, if the area ratio of liquid-solid contact is *f*, then the area ratio of liquid-air contact is 1 − *f*. The Cassie equation can be described as:

(4) $$\cos\theta^{*}=f\cos\theta +(1-f)\cos180{}^{\circ}=f(\cos\theta +1)-1$$

It is revealed that liquid will fill into the rough microstructures in the Wenzel model. Thus, the surface shows ultrahigh liquid adhesion because of the large contact area between liquid and solid surfaces [87,88,89]. The liquid droplet on this surface has a high SA value. By contrast, due to the air cushion trapped between the liquid and the rough microstructure in the Cassie model, the fluid can only touch the top of the surface microstructure, so the solid surface shows ultralow adhesion to liquid [90,91,92]. The SA of a liquid droplet in the Cassie state is usually very low and even less than 10°.

## 3. Solid/Liquid Separation: Removal of Liquids Away from a Solid Material

Some materials have great liquid-repellent properties, which make various liquids keep away from them. For example, water and aqueous solution are repelled by superhydrophobic surfaces [93,94,95,96,97,98,99,100], and superoleophobic surfaces can repel oils and organic fluids [101,102,103,104,105,106,107,108,109,110]. When liquid droplets fall on these superwetting surfaces, the droplets will bounce and roll away. The liquid seems to be separated from the surface of the materials. The superhydrophobic or superoleophobic surface microstructures significantly prevent the contact between the liquids and the solid substrates. So, the liquid-repellent microstructures can separate liquids and solid materials by avoiding liquid adhering to the surface of a material.

### 3.1. Superhydrophobicity

**Figure 3:**
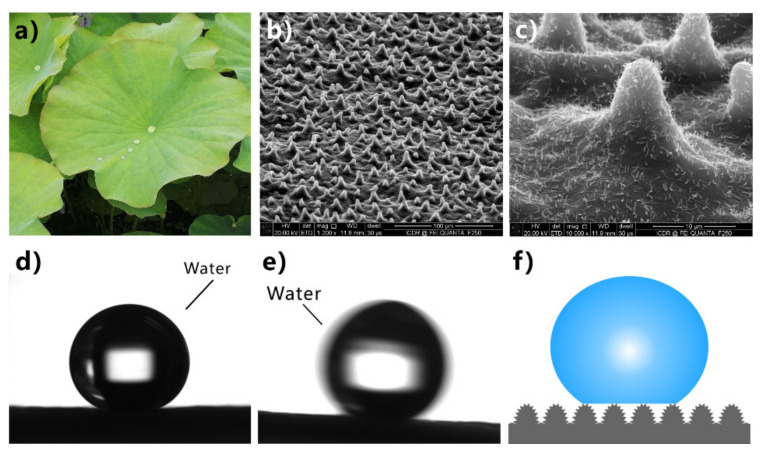
Superhydrophobicity of lotus leaves. (**a**) Lotus leaves. (**b**,**c**) Scanning electronic microscopy (SEM) images of the surface microstructure of the lotus leaf. (**d**) A drop of water on the lotus leaf. (**e**) Rolling of a water droplet on the surface of a lotus leaf. (**f**) Schematic diagram of the contact state between a water droplet and the superhydrophobic lotus leaf. Reprinted with permission from ref. [46]. Copyright 2017, American Chemical Society.

Many animals and plants have evolved superhydrophobic surfaces to adapt to their living environment [41,42,111,112,113,114,115,116,117,118,119,120,121,122,123]. The lotus leaves rise unstained from the mud because they have a great ability to repel water (Figure 3a). Such a superwetting phenomenon is known as superhydrophobicity [41,42,43,44,45,46,47]. Dewdrops can curl up into a sphere and roll back and forth on a lotus leaf. A small drop of water has a water CA (WCA) of 153° ± 2° (Figure 3d) on the lotus leaf and can easily roll away when the leaf is slightly tilted or shaken with a water SA (WSA) of ~4.5° (Figure 3e) [45,46]. The leaf is covered by a mass of microscale papilla structures (Figure 3b) [41]. The papillae have a diameter of 5~9 μm and distribute randomly. In addition, abundant nanoscale rods with a diameter of ~120 nm are distributed on the whole surface of each papilla (Figure 3c) [42]. In chemistry, the surface of the lotus leaf is covered with a thin layer of waxy crystals, which is a typical low-surface-energy material. For a water droplet on the lotus leaf, an air cushion forms underneath the water droplet, which is trapped in the rough surface microstructure of the lotus leaf. Such an air layer allows water on the lotus leaf to be at the Cassie state; that is, the water touches the top portion of the hierarchical microstructure on the lotus leaf (Figure 3f) [26,36,82,83]. Because the hierarchical surface microstructure dramatically reduces the contact area between the lotus leaf and water, the lotus leaf shows an obvious repulsion to water. The synergistic effect of the hierarchical microstructure and the hydrophobic waxy crystals with low surface energy results in the superhydrophobicity for lotus leaf [41,42,43,44,45,46,47]. Inspired by the lotus leaves, a variety of superhydrophobic materials can be prepared by combining hierarchical microstructure and low-surface-energy chemical components [124,125,126,127,128,129,130,131,132,133,134,135,136,137,138,139,140].

Superhydrophobicity is the synergistic effect between surface microstructures and low-surface-energy chemical composition. It can be obtained by directly forming micro/nanostructures on a hydrophobic substrate or structuring a hydrophilic substrate combined with hydrophobic modification.

**Figure 4:**
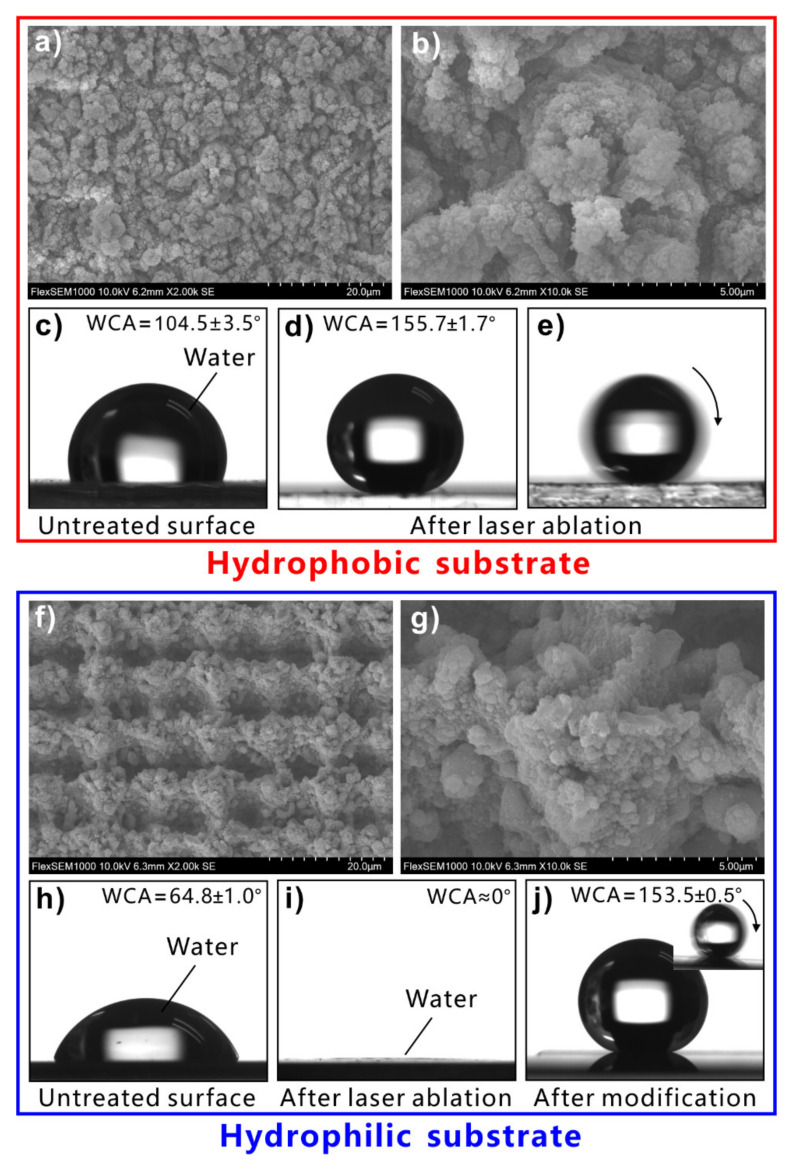
Achievement of superhydrophobicity on (**a**–**e**) hydrophobic PDMS substrate and (**f**–**j**) hydrophilic silicon substrate. (**a**,**b**) Hierarchical microstructures prepared by laser processing on the PDMS surface. (**c**,**d**) A drop of water on (**c**) the untreated flat PDMS substrate and (**d**) the structured PDMS surface. (**e**) Water droplet rolling away on the structured PDMS. (**f**,**g**) Hierarchical microstructures prepared by laser processing on the silicon surface. (**h**–**j**) A drop of water on (**h**) the untreated flat silicon surface, (**i**) the structured silicon surface, and (**j**) the structured silicon surface modified with low-surface-energy chemistry. The inset in (**j**) shows the rolling process of a water droplet on the resultant silicon surface. Reprinted with permission from ref. [151]. Copyright 2020, WILEY-VCH Verlag GmbH & Co. KGaA.

Regarding the intrinsically hydrophobic substrates with low surface energy, the superhydrophobicity can be obtained by directly building hierarchical microstructures on the material surface [141,142,143,144,145,146,147,148,149,150]. For example, polydimethylsiloxane (PDMS) is inherently hydrophobic because it has rich low-surface-energy –CH_2_ and –CH_3_ groups. Water droplets on the untreated flat PDMS surface have a WCA of 104.5°–110° (Figure 4c). Yong et al. produced numerous coral-like surface microstructures on a PDMS substrate via femtosecond laser microfabrication (Figure 4a,b) [151]. The size of the coral-like structures is only about several micrometers. The surface of the microscale corals is decorated with a large number of nanoscale protrusions. Without any chemical modification, the laser-ablated PDMS surface exhibits superhydrophobicity due to the formation of the micro/nanoscale hierarchical structures. The WCA of a water droplet on the rough PDMS surface is 155.7° ± 1.7° (Figure 4d). The droplet can easily roll away with a WSA of 1° (Figure 4e).

However, simply relying on the surface microstructure is not enough to obtain superhydrophobicity on a hydrophilic substrate. For an inherently hydrophilic substrate, the surface energy must be further lowered to achieve superhydrophobicity besides the formation of rough surface microstructures [152,153,154,155,156,157,158,159,160,161]. The structured surface can be modified with the chemical layer with low surface energy. For example, silicon is a hydrophilic material, with a WCA of 64.8° ± 1.0° to water droplets (Figure 4h). The hierarchical microstructures can also be easily prepared on the silicon substrate by femtosecond laser ablation (Figure 4f,g) [151]. There are mountain-like structures periodically distributed on the structured silicon surface. The mountains are about 2.9 μm in height and about 6 μm in diameter. The surface of the micromountains is covered with nanoscale particles, which form a hierarchical microstructure. Water can sufficiently wet the rough silicon surface, resulting in a final WCA near 0° (Figure 4i). So, the rough silicon surface is superhydrophilic. After modifying the rough silicon surface with fluoroalkylsilane, the wettability of the silicon sample switched to superhydrophobic. The water droplets on the surface of the modified silicon are spherical with a WCA of 153.5° ± 0.5° (Figure 4j), which roll off easily under slight vibration (inset of Figure 4j).

The formation of hierarchical microstructure is the key to achieving superhydrophobicity. For a hydrophilic substrate, rough microstructures should be created on the material surface first. The surface further needs to be modified with the low-surface-energy molecular layer to obtain superhydrophobicity. If the substrate is an intrinsically hydrophobic material, superhydrophobicity can be directly achieved by constructing proper rough microstructures on the material surface.

### 3.2. Superoleophobicity

**Figure 5:**
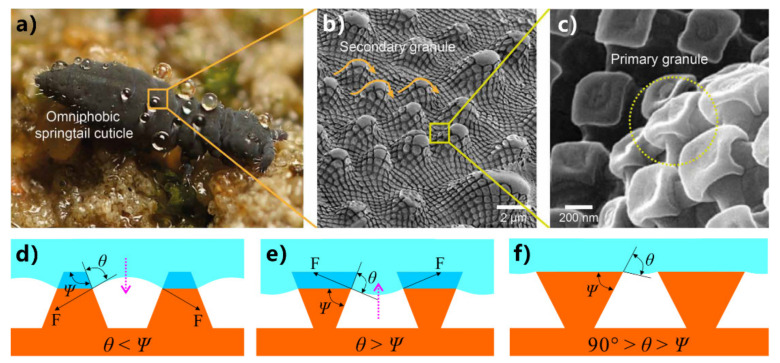
Liquid repellency of a springtail. (**a**) Photograph of a springtail. (**b**,**c**) Surface microstructure of springtail skin. Reprinted from ref. [163]. (**d**–**f**) Importance of the re-entrant structure in the achievement of in-air superoleophobicity: (**d**,**e**) hypothetical solid-air-liquid interfaces on (**d**) a trapezoid texture and (**e**) an inverted-trapezoid texture, and (**f**) robust Cassie state of liquid with low surface tension on the inverted-trapezoid (re-entrant) texture. Reprinted from ref. [36].

Because the surface tension of organic liquids is much lower than that of water, ordinary superhydrophobic surfaces are usually wetted with oil [36,162]. Interestingly, Springtails (Figure 5a) have the ability to repel organic liquids with low surface tension [163,164,165]. Springtails often live in a habitat with rich surface-active ingredients, where water is usually polluted by decayed organic matter. Besides superhydrophobicity, the springtails skin even behaves superoleophobic, preventing organic liquids from wetting the skin. A stable plastron forms on the skin of the springtail when submerged in water or even in many oil liquids because of the excellent superoleophobicity. The springtail can breathe air through this plastron layer, thus avoiding the danger of suffocation in the liquid. The skin of the springtail is entirely covered by a large number of bristles and rhombic meshes (Figure 5b) [165]. The rhombic meshes are composed of interconnected nano-granules (Figure 5c). Both the ridges and granules have a negative overhang shape, which is a typical re-entrant structure. It is demonstrated that such re-entrant microstructure enables the springtail skin to have anti-oil ability. The unit of the re-entrant microstructure usually consists of a large flat top and a thin support rod.

It is much more difficult to prepare a superoleophobic surface than a superhydrophobic surface as the surface tension of oil is much smaller than the surface tension of water [36]. The preparation of a superoleophobic surface in the air usually requires re-entrant structure and strict chemical modification to reduce the surface energy, in addition to having a sufficiently rough micro/nanostructure [29,166,167,168,169,170,171,172,173,174,175,176,177,178,179,180]. On a superoleophobic surface, even oil droplets with a surface tension of 20~30 mN/m can have an oil CA (OCA) of 150° or greater. Tuteja et al. first pointed out the importance of the re-entrant curvature structure for achieving superoleophobicity in the air [168]. On any flat surface, droplets with very low surface tension (such as oils and organic solvents) have Young’s CA of much less than 90°. The solid-air-liquid interface can be divided into two cases when the liquid with ultralow surface tension (*θ* < 90°) is assumed to contact two different textured surfaces, as shown in Figure 5d,e [36]. It is assumed that two substrates are composed of the same material and therefore have the same surface energy. The local geometric angle of the texture is labeled as *ψ*. In the case of *θ* < *ψ* (Figure 5d), the net traction at the liquid-gas interface is downward, promoting the liquid to penetrate the solid texture. As a result, the rough microstructures of the solid texture are fully wetted by the liquid. However, when *θ* > *ψ* (Figure 5e), the net traction is upward and can drive the liquid-gas interface back to the top of the columns, thus forming a composite solid-liquid-gas interface (Figure 5f). Therefore, there is a stable Cassie state only for *θ* ≥ *ψ* [36,168,169,181]. Based on this design constraint, many trapezoid-shaped superhydrophobic microstructures (*ψ* > 90°) are unable to support low-surface-tension liquids to form a composite interface (at robust Cassie state). Only the inverted trapezoid-shaped surfaces (*ψ* < 90°) have re-entrant texture, which provides the possibility of oil in the Cassie state on a solid substrate (Figure 5f). It is revealed that re-entrant textures are necessary for developing superoleophobic surfaces [168,169,181]. However, the standard re-entrant structure can only be fabricated in a limited number of ways, such as electron beam etching and 3D printing. Usually, similar re-entrant structures with overhang, inverted trapezoid, and mushroom-like shapes have been developed to obtain superoleophobicity in the air [29,36,166,167,168,169,170,171,172,173,174,175,176,177,178,179,180].

**Figure 6:**
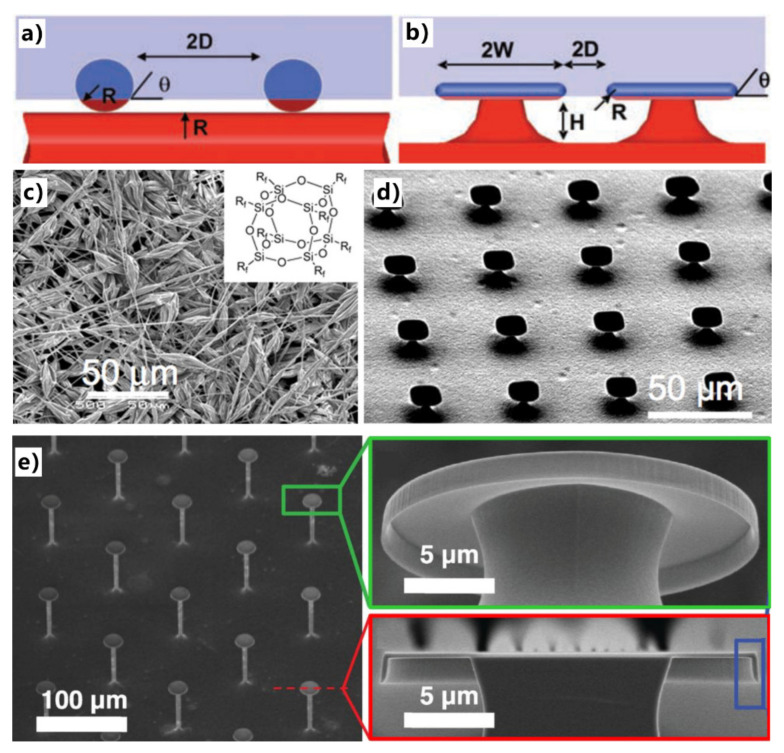
Superoleophobic surfaces prepared by constructing re-entrant curvature. (**a**,**b**) Schematic diagram of the re-entrant topography of the (**a**) fibers and (**b**) micro-hoodoos. (**c**) Morphology of the electrospun fibers. Inset: the structure of POSS molecule. (**d**) Morphology of the microhoodoo surface having square and circular flat caps. Reprinted with permission from ref. [168]. Copyright 2007, The American Association for the Advancement of Science. (**e**) SEM images of the doubly re-entrant micro-posts with vertical overhang. These superoleophobic surfaces possess re-entrant microstructures. Reprinted with permission from ref. [182]. Copyright 2014, The American Association for the Advancement of Science.

Tuteja et al. designed and fabricated two types of re-entrant-curvature microstructures [168,169]. Figure 6a,c shows the electrospun fiber mat formed by blending hydrophilic polymer (polymethyl methacrylate) with fluorodecyl polyhedral oligomeric silsesquioxane (POSS) molecules. The synthesized POSS molecules are surrounded by rich fluorinated alkyl groups (inset of Figure 6c). The “beads-on-strings” morphology of the fiber mat provides multi-scale re-entrant texture in the surface topography and significant porosity in the mat. “Microhoodoo” microstructures were also produced on a silicon substrate through SiO_2_ deposition and a subsequent etching process, as shown in Figure 6b,d. The microhoodoo has an under-cut silicon pillar and is capped with a layer of SiO_2_. Although the composition of fluorodecyl POSS on a flat substrate shows oleophilic with an intrinsic OCA small than 90°, the POSS-modified fiber mats and microhoodoo surface with re-entrant surface curvature presented superoleophobicity in air. Surprisingly, the overhanging microstructure of re-entrant even allows the oleophilic material to repel low-surface-energy oils and organic solvents. Kim et al. prepared an array of microscale posts (Figure 6e) [182]. The top of the posts has a vertically nanoscale overhanging structure, forming a doubly re-entrant feature for the posts. Without any low-surface-energy modification, the as-prepared surface could repel even liquids with ultralow surface tension (i.e., *γ* < 15 mN/m). For example, various oils, organic solvents, and even fluorinated solvents cannot wet the as-prepared post array. The solvent can wet the top of the re-entrant post structure and run down the vertical overhangs. Once the liquid reaches the bottom of the overhangs, the direction of surface tension at this location will change from downward to upward. This causes the fluid to stop penetrating downward and forms a stable liquid suspension on the micro-posts. The result reveals that the surface with a multi-level re-entrant microstructure has stronger liquid resistance than a single re-entrant structure.

**Figure 7:**
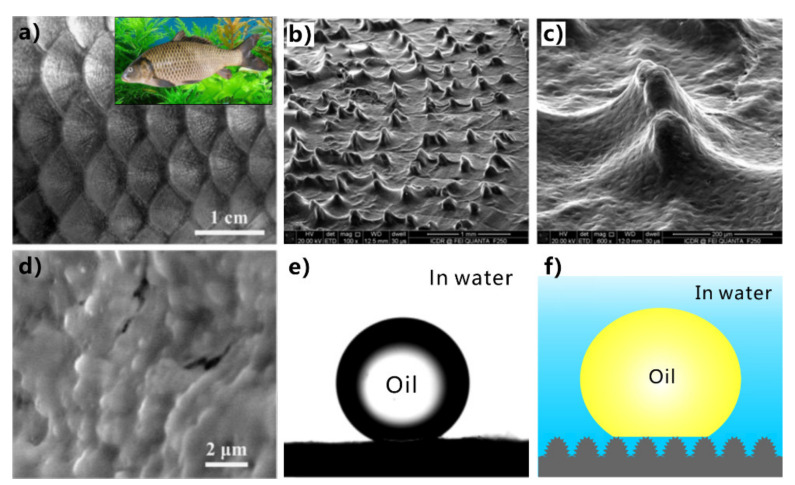
Underwater superoleophobicity of fish scales. (**a**) Photos of fish (inset) and fish scales. (**b**–**d**) Microstructures on the fish scales. (**e**) A drop of oil on the fish scale in water. (**f**) Schematic diagram of an oil droplet on the surface microstructures of the underwater superoleophobic fish scale in water. Reprinted with permission from ref. [49]. Copyright 2009, WILEY-VCH Verlag GmbH & Co. KgaA. Reprinted with permission from ref. [46]. Copyright 2017, American Chemical Society.

Oils cannot contaminate fish scale (Figure 7a) in oil-polluted water [48,49]. It is revealed that the oil resistance of fish is attributed to the superoleophobicity of its scales underwater [48]. The fish body is covered with fan-shaped scales, which are composed of hydrophilic calcium phosphate and protein and are attached to mucus. Each scale is lined with fine hill-like structures with hundreds of micrometers (Figure 7b,c) [49]. Nanoscale pimple structures also decorate on the surface of each micro-hill (Figure 7d). Underwater oil droplets remain spherical on the fish scales with an OCA of 151.5° ± 2° (Figure 7e) and are easy to roll away, indicating the excellent underwater superoleophobicity of the fish scales. As shown in Figure 7f, the surface microstructures of fish scales are wetted by water in a water medium. When the oil droplets are dispensed onto the fish scales, there is a layer of water underneath the oil. The trapped water cushion prevents oil from effectively contacting the microstructure of the scale surface, so the fish scales repel oils in the water. The contact of the oil on the fish scale is consistent with the underwater Cassie state (Figure 7f) [30,36,162]. The underwater superoleophobicity of fish scales results from the rough surface microstructures and the chemical composition with high surface energy. Underwater superoleophobic surfaces can be easily prepared based on the effective route of “from in-air superhydrophilicity to underwater superoleophobicity”.

Inspired by fish scale, underwater superoleophobicity can be obtained by producing rough microstructures on a hydrophilic substrate [183,184,185,186,187,188,189,190,191,192,193,194,195,196,197,198,199,200]. For example, the hydrogel is hydrophilic and can keep water molecules. Using polyacrylamide (PAM) hydrogel as a copy material, Liu et al. obtained underwater superoleophobicity on the hydrogel substrate by directly replicating fish scales [48]. The PAM replica has the same surface microstructure as the fish scales and shows an OCA of 162.6° ± 1.8° to oil droplets in water. Silicon is also inherently hydrophilic, and the WCA of a water droplet on the smooth silicon surface is 52.5° ± 1.4°. Liu et al. prepared microscale and nanoscale structures on this hydrophilic silicon substrate by lithography method [48]. The silicon surface with micro/nanoscale hierarchical structure is superoleophobic to 1,2-dichloroethane droplets with an OCA of 174.8° ± 2.3° underwater. The adhesive force between the surface of the underwater superoleophobic silicon and the oil droplets is less than 1 mN, making it difficult for the oil droplets to stay on the surface stably. The as-prepared underwater superoleophobic surfaces can repel all kinds of oil such as 1,6-dibromohexane, octane, 1-bromo-n-octane, 1,1,2,2-tetrabromoethane, and 1-bromododecane.

**Figure 8:**
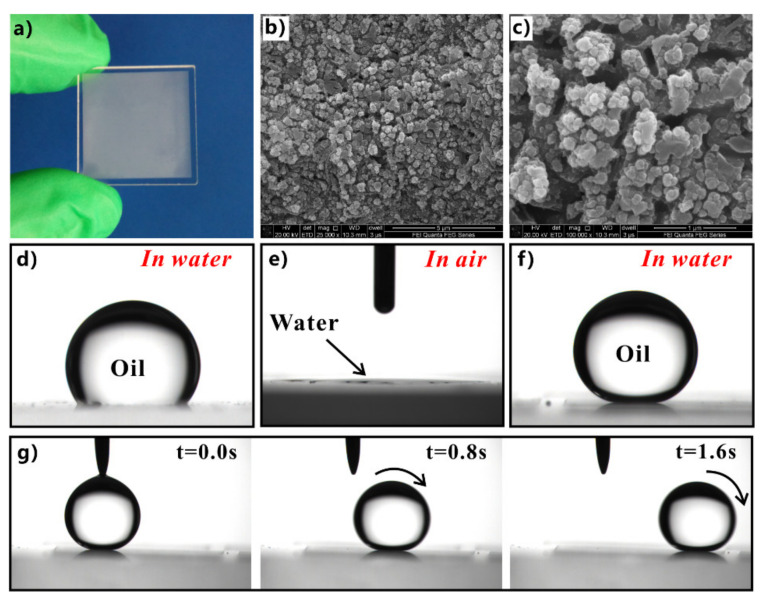
Achievement of underwater superoleophobicity on the silica glass substrate by forming surface microstructure. (**a**) Photo of the structured glass sheet. (**b**,**c**) Nanostructure on the glass surface. (**d**) A drop of oil on the original flat glass surface underwater. (**e**) Water wetting the structured glass surface in the air. (**f**) Underwater oil droplet on the structured glass surface. (**g**) The rolling process of an oil droplet on the sample surface with a small tilted angle in the water. Reprinted with permission from ref. [201]. Copyright 2015, The Royal Society of Chemistry.

The silica glass is intrinsically hydrophilic with a WCA of 33.3°. The original silica glass exhibits weak oleophobicity in a water medium, with an OCA of 125.5° to oil droplets (Figure 8d). Yong et al. produced a rough nanoscale structure on the surface of silica glass using femtosecond laser processing (Figure 8a) [201]. The resultant surface is covered with a large number of irregular particles with sizes ranging from tens to hundreds of nanometers (Figure 8b,c). Water droplets can spread out completely on the surface of the structured glass (Figure 8e). The hydrophilicity of the glass substrate is amplified to superhydrophilicity by the generated rough microstructure. When the superhydrophilic rough glass is immersed in water, the oil droplets on the glass surface could remain spherical, with an OCA of 160.2° (Figure 8f). Once the glass surface is tilted by 1°, the oil droplets will quickly roll away (Figure 8g). Therefore, the formation of microstructures makes the glass surface exhibit underwater superoleophobicity to oils in water.

The in-air superoleophobicity and underwater superoleophobicity are two completely different systems. Their formation mechanism is different, and the application situation is also different. The re-entrant microstructure is the key to realizing superoleophobicity in the air. Unlike the preparation of in-air superoleophobic surfaces, underwater superoleophobic surfaces can be easily fabricated by generating microstructure on a hydrophilic substrate. Both types of superoleophobic surfaces have a strong repellence to oils and oily liquids, but the former materials work in the air while the underwater superoleophobic materials work in the water.

### 3.3. Liquid Repellence

Superhydrophobic surface and superoleophobic surface have excellent water- or oil-repellent ability. The liquid repellence of the superwetting surfaces enables liquids to be difficult to adhere to such a solid material. The result looks as if the liquids are separated from the surface of the materials.

**Figure 9:**
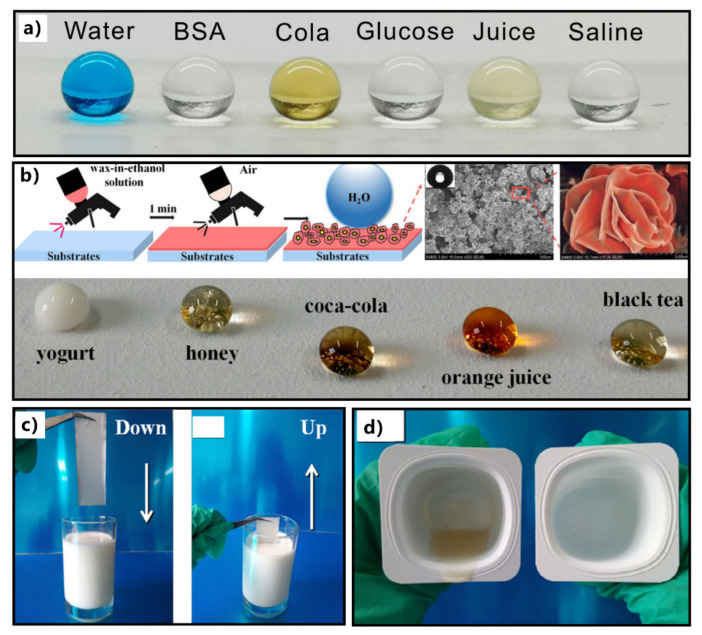
Liquid repellence of the artificial superwetting surfaces. (**a**) Repellence of the hierarchical porous PTFE surface to various liquids. (**b**) Fabrication of superhydrophobic coating resistant to a variety of liquids. (**c**) The superhydrophobic coating thrusting into the yogurt followed by an upward thrust motion. (**d**) After tipping viscous food honey out of (left) an uncoated cup and (right) a superhydrophobic cup. Reprinted from ref. [202]. Reprinted with permission from ref. [203]. Copyright 2019, Elsevier.

Xi et al. used one-step femtosecond laser ablation to create hierarchical micro/nanostructures and porous microstructure on the surface of polytetrafluoroethylene (PTFE) [202]. The micropores provide a specific re-entrant curvature. The resultant surface can repel various pure and complex liquids, such as water, cola, juice, glucose, saline, and bovine serum albumin (BSA) (Figure 9a). Liu et al. sprayed the hot ethanol solution of rice bran wax and edible candelilla wax onto various substrates (e.g., tinfoil, PTFE film, polypropylene, paper, and glass slide) and obtained a kind of superhydrophobic coating (Figure 9b) [203]. The self-roughed beautiful “flower-like” microstructure combined with the low surface energy of the wax gives these solid substrates superhydrophobicity. This low-cost coating is remarkably resistant to a variety of non-Newtonian viscous food liquids (e.g., yogurt, honey, and egg white) or hot water solutions (Figure 9b). The droplets of these liquids or solutions have CAs higher than 150° and SAs close to 0° on the superhydrophobic surfaces. When the superhydrophobic surface was inserted into the yogurt and then taken out, the surface remained clean without any yogurt adhesion (Figure 9c). The liquid food residue could be reduced and even eliminated by spraying the superhydrophobic coating on the inner surface of the food packing box (Figure 9d).

**Figure 10:**
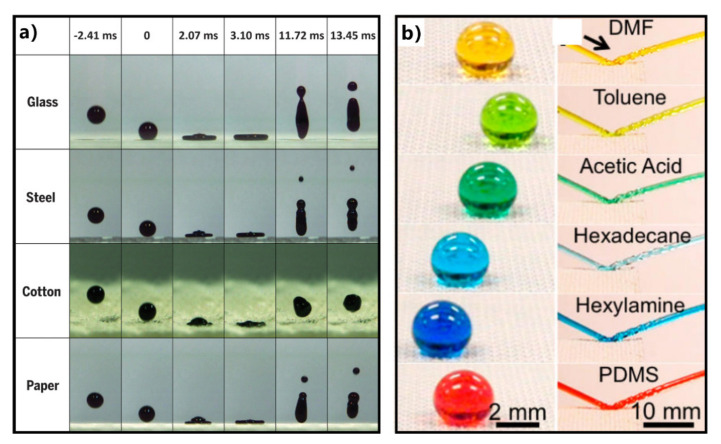
(**a**) Re-bounce of the water droplets on different superhydrophobic-coated substrates. (**b**) Jets of different Newtonian liquids rebounding on the superamphiphobic mesh. Reprinted with permission from ref. [204]. Copyright 2015, The American Association for the Advancement of Science. Reprinted with permission from ref. [205]. Copyright 2013, American Chemical Society.

Lu et al. prepared an ethanol-based suspension of titanium dioxide nanoparticles [204]. The nanoparticles were modified with perfluorosilane and had a dual-scale structure. Such suspension coating could be sprayed, dipped, or painted onto most soft and hard substrates. The coated surfaces show stable superhydrophobicity and have a resilient water repellence. On the as-prepared water-repellent surface, falling water droplets bounce off rather than infiltrate the surface. Figure 10a shows the dropping test results of water droplets on the surface of the superhydrophobic-coated glass, steel, cotton wool, and filter paper. Upon contact with these surfaces, the droplets could rebound and leave substrates completely without wetting or even contaminating these materials. Pan et al. electrospinned a mixture of crosslinked PDMS and 50 wt% fluorodecyl POSS onto a stainless-steel mesh to obtain a superamphiphobic surface (with both superhydrophobicity and superoleophobicity) [205]. The generated microstructure with hierarchical morphology has re-entrant curvature. The droplets of low-surface-tension Newtonian liquids with different polarity and non-polarity show high apparent CAs on the superamphiphobic surface (Figure 10b). Interestingly, even liquids with surface tension less than 25 mN/m have a low CA hysteresis and roll-off angle on the as-prepared surface. The formation of superamphiphobicity cannot be separated from the re-entrant curvature of the surface microstructure and the ultralow surface energy of the coating. The excellent liquid repellence enables jets of a range of Newtonian liquids (such as dimethylformamide (DMF), toluene, acetic acid, hexadecane, hexylamine, and PDMS) to rebound on the superamphiphobic mesh (Figure 10b). As the liquids bounce off the solid surface, they seem to be separated from the solid materials.

### 3.4. Connotation of Liquid Repellence in Liquid/Solid Separation

The liquids cannot wet the superhydrophobic and superoleophobic materials. The superhydrophobic/superoleophobic microstructures break the continuous contact between liquids and material surfaces at the solid-liquid interfaces, resulting in the liquids being cut off from the solid materials. The surfaces are endowed with a great ability to repel liquids. The liquid-repellent surface microstructures such as a separation film, making the liquids away from a solid material. Therefore, the superwetting microstructure can achieve solid/liquid separation.

We only briefly point out the connotation of the liquid-repellent surfaces (e.g., superhydrophobic surfaces and superoleophobic surfaces) in the aspect of solid/liquid separation here, but will not show too many superhydrophobic and superoleophobic examples because a large number of review articles on the superhydrophobicity and superoleophobicity have been published over the past two decades [26,27,28,29,30,31,32,33,34,36,37,38,39,40,124,125,126,127,128,129,130,131,132,133,134,135,166,167,181]. The liquid-repellent materials can be designed by combining appropriate micro/nanoscale surface morphology and chemical composition. All of the developed micro/nano machining methods can produce superwetting structures on a solid substrate. Because of the excellent liquid repellence, the superhydrophobic and superoleophobic materials have rich practical applications in anti-liquids [36], self-cleaning [206,207,208,209], anti-icing/fogging/snowing [210,211,212], antifouling [82], anticorrosion [205,213], drag reduction [214], water harvesting [215], manipulation of liquid droplets [216,217,218], liquid patterning [219,220], microfluidics [221,222], lab chip [223,224], cell engineering [225,226], buoyancy enhancement [47,227], and so on.

## 4. Oil/Water Separation Based on Superhydrophobic or Underwater Superoleophobic Materials

With the continuous growth of the demand for global energy, accidents such as oil spills and industrial discharges of oily wastewater frequently occur [10,228]. For example, about 210 million gallons of oil were released on the ocean surface in the Gulf of Mexico spill in 2010 [229]. These alarming oil spills caused huge economic loss and seriously destroyed the ecological environment. The above accidents should be avoided as far as possible. On the other hand, advanced oil/water separation technology should be developed to deal with the increasing oil pollution problem and respond to emergencies. Traditional separation technology includes gravity separation, centrifugal separation, flotation, skimming, etc. [4,10,19]. Although these technologies can also play a certain role in separating oil/water mixtures, they do not have selective separation or absorption capacity with high efficiency. The purity of the separated oil could not meet the requirements of secondary use. In addition, the traditional separation materials cannot resist oil pollution, making most of these separation materials are disposable materials. At present, the preparation of a new type of recyclable oil/water separation material with filtering and absorbing selectivity to oil and water has become one of the hot research topics. To separate the oil/water mixture, the oil phase and the water phase must be subjected to opposite effects. Interestingly, many materials (e.g., superhydrophobic-superoleophilic or superhydrophilic-superoleophobic materials) show opposite superwettabilities to water and oil; that is, they have different interface effects for water or oil. The opposite wettabilities of the materials to water and oil can provide the basis for separating the mixture of oil and water. Because of their different wettabilities to water and oil, superhydrophobic/superoleophilic or underwater superoleophobic materials have been successfully used to separate the mixtures of water and oils in the past decades [1,4,10,17,19,20].

### 4.1. Superhydrophobic Porous Mesh/Membranes

**Figure 11:**
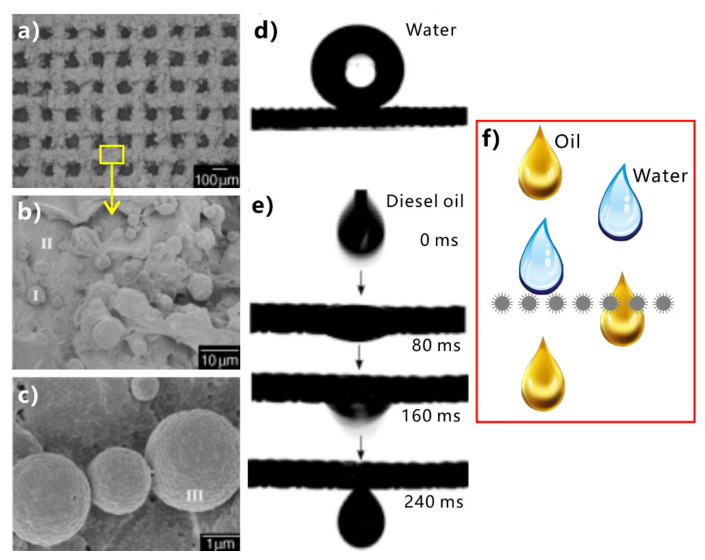
Separating the mixture of oil and water by using the superhydrophobic PTFE-coated stainless-steel mesh. (**a**–**c**) Surface morphology of the structured mesh. (**d**) Water droplet on the PTFE-coated mesh. (**e**) Process of oil droplets permeating through the resultant mesh. (**f**) Schematic separation mechanism for the oil/water mixture by the superhydrophobic and superoleophilic porous mesh. Reprinted with permission from ref. [61]. Copyright 2004, WILEY-VCH Verlag GmbH & Co. KgaA.

Superhydrophobic/superoleophilic porous mesh/membrane is a typical “oil-removing” material. Such material can allow oils to pass through freely, but the water is excluded, so this type of material can separate the mixture of oil and water [230,231,232,233,234,235,236,237,238,239,240]. In 2004, Feng et al. prepared a PTFE-coated metal mesh and used this mesh to successfully separate the oil/water mixture (Figure 11) [61]. The superhydrophobic mesh was fabricated by a simple spray-and-dry process. The homogeneous emulsion of hydrophobic PTFE was sprayed evenly onto the surface of a stainless-steel mesh with a pore diameter of ~115 µm. After heating, a layer of rough PTFE structure formed on the mesh surface (Figure 11a). Abundant balls with sizes ranging from 2 to 5 µm are evenly distributed on the coating (Figure 11b). The surface of each ball is further decorated with dense craters with a diameter of about 71 nm (Figure 11c). Water droplet has a WCA of 156.2° ± 2.8° and a WSA of 4° on the PTFE-coated mesh (Figure 11d). When the droplets of diesel oil were constantly dropped on this mesh, they would quickly wet the mesh and permeate through within 240 ms (Figure 11e). The results indicate that the rough mesh is superhydrophobic to water and superoleophilic to oils. To achieve oil/water separation, the mixture of oil and water was poured on the as-prepared superwetting metal mesh. The superhydrophobicity ensures that the water is intercepted and maintained above the mesh. By contrast, the superoleophilicity allows the oil to quickly wet the mesh and further permeate through (Figure 11f). The mixture of oil and water was successfully separated.

**Figure 12:**
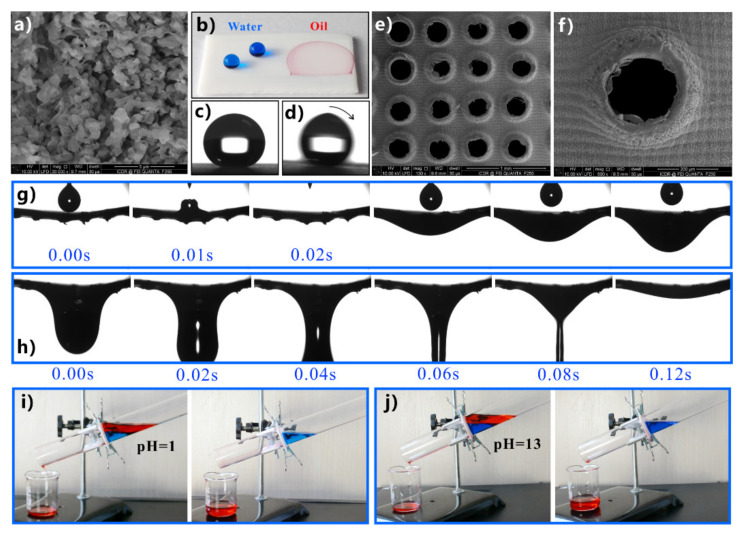
Achievement of oil/water separation by the laser-induced superhydrophobic/superoleophilic porous PTFE sheet. (**a**) Microstructure of the laser-ablated PTFE surface. (**b**) Photo of the drops of water and oil on the structured PTFE surface. (**c**) Static profile and (**d**) rolling snapshot of a small water droplet on the laser-ablated sheet. (**e**,**f**) Morphology of the structured porous PTFE sheet. (**g**) Oil droplets rapidly permeating through the porous sheet and (**h**) dripping down. (**i**) The separation of the mixture of strong acid solution and oil and (**j**) the separation of the mixture of strong alkaline solution and oil by the porous, rough PTFE sheet. Water was dyed blue, and oil was dyed red. Reprinted with permission from ref. [241]. Copyright 2019, Elsevier.

Yong et al. fabricated hierarchical microstructures on the surface of a PTFE sheet by femtosecond laser processing (Figure 12a) [241]. The water droplet on the structured sheet has a WCA of 155.5° (Figure 12b,c) and can easily roll away (Figure 12d). The oil droplet on the structured sheet has a very small OCA of approximately 0° (Figure 12b). The results indicate that the structured PTFE sheet is superhydrophobic and superoleophilic at the same time. A series of perforations were further formed in the PTFE sheet through mechanical drilling (Figure 12e,f). When drops of oil fell on the as-prepared porous PTFE sheet, the oil rapidly passed through the microholes and dripped down (Figure 12g,h). The as-prepared porous PTFE sheet could act as a separation membrane to achieve oil/water separation. Even the mixtures of strong acid or strong base solution and oil could be successfully separated (Figure 12i,j). When the mixture of water and strongly acidic/alkaline liquid was poured into the designed separator, the oil could thoroughly permeate through the separation membrane. In contrast, the water was repelled by the sheet and stayed above the PTFE sheet. Thus, the oil was removed from the strong acid/base aqueous solution.

**Figure 13:**
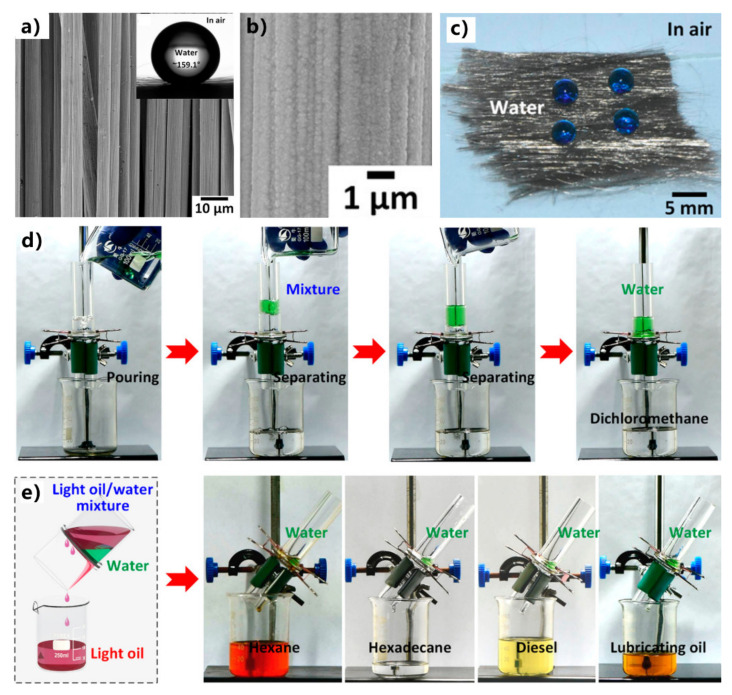
Oil/water separation based on the superhydrophobic nickel-coated carbon fiber. (**a**,**b**) Microstructure of the superhydrophobic carbon fiber coated with nanoscale nickel grains. Inset of (**a**): Shape of a water droplet on the as-prepared carbon fibers. (**c**) Photo of water droplets on the as-prepared carbon-fiber sheet. (**d**) Separation of water and heavy oils (such as dichloromethane). (**e**) Separation of the water and light oils (hexane, hexadecane, diesel, and lubricating oil). Reprinted with permission from ref. [242]. Copyright 2020, American Chemical Society.

Huang et al. obtained superhydrophobicity on a carbon-fiber sheet by nickel electroplating [242]. A layer (thickness = ∼333 nm) of nanoscale nickel grains was uniformly deposited onto the surface of the entire microscale fiber, resulting in micro/nanoscale structures (Figure 13a,b). After fluoroalkylsilane modification, the nickel-coated carbon fibers are superhydrophobic, with a WCA of ∼159.1° and a WSA of ~12.6° (inset of Figure 13a,c). The as-prepared carbon fiber also shows superoleophilicity to oil droplets. To achieve oil/water separation, two individual carbon fibers were placed vertically and were sandwiched between two tubes. For the mixtures of water and heavy oils (such as dichloromethane), the separation equipment was vertically placed (Figure 13d). Water was unable to penetrate the fibers. The higher density allowed the heavy oil to sink to the bottom position (below water), and the superoleophilicity of the fibers enabled the oil to permeate through the fibers finally. Therefore, the heavy oil and water were separated. In the case of the separation of light oils (such as hexane, hexadecane, diesel, and lubricating oil) and water, the separation equipment should be inclined to ensure the contact between oils and the fibers (Figure 13e). The separation efficiency of the superhydrophobic nickel-coated carbon fibers was higher than 99.1% when applied for either heavy or light oil. The oil content in the separated water was less than 78 ppm, indicating that the collected oils and water had a very high purity.

Wang et al. deposited a film of rough copper on the wire surface of a copper mesh through the electrochemical deposition method [243]. After being modified by a long-chain fatty acid, the rough mesh became superhydrophobic and superoleophilic. Water droplets on the mesh had a WCA of 158° ± 2.1°, while diesel oil droplets could permeate through the mesh freely. Such a copper mesh separated the mixture of water and diesel oil. Li et al. burned a metal mesh by the burning flame from paraffin [244]. As a result, the surface of the mesh was wrapped with a layer of carbon nanoparticles. The smaller hydrophobic silica particles were further sprayed onto the mesh surface with high-pressure gas. The resultant mesh had stable superhydrophobicity and superoleophilicity and could resist water, acid, and alkali corrosion. The opposite wettability to water and oil endows the as-prepared mesh with the ability to separate a variety of oil/water mixtures because the mesh only allows oils to pass through. Baig et al. synthesized cerium oxide nanoparticles of high purity by a simple co-precipitation method and spray-coated the nanoparticles onto the surface of a stainless-steel mesh [245]. The formation of the uniform coating turned the microporous membrane to superhydrophobic and superoleophilic. The coated mesh has two functions. On the one hand, the superwettability enables the mesh to separate the oil/water mixtures with high efficiency (~99%). The oil passed through the mesh rapidly while water was blocked. On the other hand, the coated mesh can quickly and effectively degrade harmful environmental pollutants through the photocatalytic process of UV light irradiation.

Zhou et al. incorporated the polyaniline and fluorinated alkylsilane into the cotton fabric through in situ vapor phase deposition [246]. Thus, a layer of the hydrophobic nanocomposite structure was produced on the surface of each textile fiber. The coating led to superhydrophobicity and superoleophilicity for the fabric, with a WCA of 156° and an OCA of 0°. The superhydrophobic fabric repelled water but allowed the permeation of oil. So, the oil could be successfully separated from the mixture of water and oils. The fabric exhibited stable recyclability during the repeated separation and maintained high efficiency in various harsh conditions. Dong et al. constructed superhydrophobic coatings on different fabrics through an eco-friendly strategy [247]. The desired surface microstructure was created on the surface of the fabrics by coating polydopamine on the fabrics. After being modified with the low-surface-energy stearic acid, the fabrics finally showed superhydrophobicity with a WCA of 162.0° and a WSA of 7.8°. The water and heavy oil mixture could be easily separated by using the superhydrophobic fabrics as a selective filter membrane. The gravity force could only drive the heavy oil to penetrate the fabric, but the water was blocked because of the water repellence of the superhydrophobic fabric. Ma et al. added silica nanoparticles to electrospun polyimide nanofibers and prepared a fluorine-free, superhydrophobic, and superoleophilic nanofiber membrane through in situ polymerization [248]. Oil could permeate through the membrane, but the water was blocked, which allowed the membrane to separate oil and water effectively. The membrane had a high separation efficiency of 99%, a high separation speed of 4798 L m^−2^ h^−1^, and suitable reusability toward the oil/water separation application. After various oil/water mixtures were separated, the oil content was less than 5 ppm in the collected water. In addition, the membrane has exceptional thermal and chemical stability and can be resistant to the high-concentration salt solution. Hou et al. obtained the superhydrophobic dispersion of poly[(3,3,3-trifluoropropyl)methylsiloxane] aggregations through the phase separation process [249]. When the aggregations were coated on a non-woven fabric, the fabric was endowed with superhydrophobicity. Using such a superhydrophobic fabric as a filtrate membrane, the mixture of water and kerosene was successfully separated.

Zhang et al. formed rough structures with different scales on the surface of a melamine sponge through dry-wet chemical methods [250]. As the sponge was further coated with a layer of PDMS, the sponge possessed superhydrophobicity and superoleophilicity. Oil could quickly pass through the superoleophilic sponge, while the superhydrophobicity prevented water from penetrating, thus separating water and oils.

### 4.2. Underwater Superoleophobic Porous Mesh/Membranes

Superhydrophobic “oil-removing” materials are easily blocked in the separation process because the oil adheres to the filter membrane as it passes through. This decreases the separation efficiency achieved and the service life of the materials. Furthermore, the water settles below the oil in the mixture of water and light oils (which are less dense than water). A layer of water forms between the oil and the filter membrane, which prevents the oil from maintaining contact with the membrane. Therefore, the superhydrophobic membrane is not suitable for the gravity-driven removal of light oils from mixed solutions. Unfortunately, most oils are lighter than water. “Water-removing” superhydrophilic and superoleophobic materials have been developed to address these problems.

**Figure 14:**
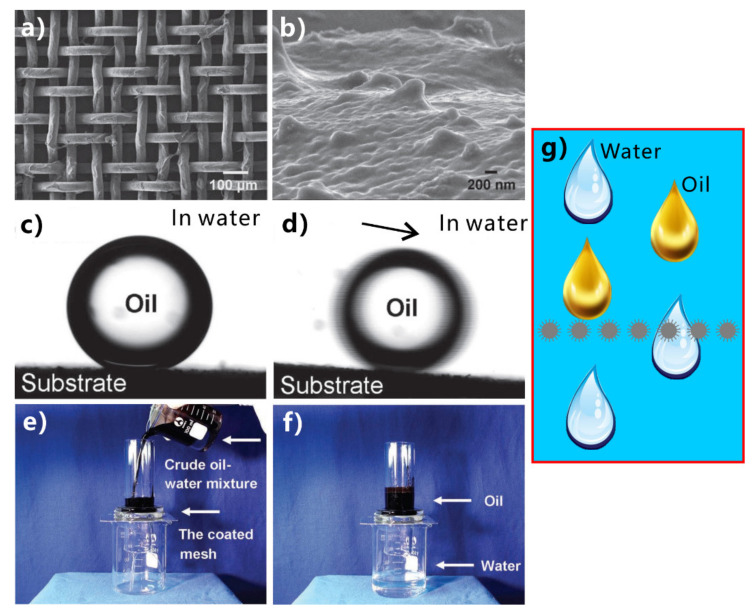
Separating the mixture of oil and water by the underwater superoleophobic stainless-steel mesh coated with hydrogel. (**a**,**b**) Surface morphology of the hydrogel-coated mesh. (**c**) A drop of oil on the hydrogel-coated mesh in water. (**d**) Rolling of an underwater oil droplet on the resultant mesh. (**e**,**f**) Separation of the mixture of crude oil and water by the prewetted mesh: (**e**) pouring the mixture onto the separation mesh and (**f**) after separation. (**g**) Schematic diagram of the separation mechanism based on the superhydrophilic and underwater superoleophobic porous mesh. Reprinted with permission from ref. [62]. Copyright 2011, WILEY-VCH Verlag GmbH & Co. KgaA.

Inspired by the underwater oil resistance of fish scales in water, various underwater superoleophobic materials have been developed by designing rough micro/nanostructures on the hydrophilic substrates. Such materials are usually superhydrophilic in air and have oil repellence underwater. The underwater superoleophobic materials have also been successfully applied in oil/water separation [251,252,253,254,255,256,257,258,259,260,261]. In 2011, Xue et al. obtained an underwater superoleophobic mesh by coating hydrophilic polyacrylamide hydrogel on a stainless-steel mesh [62]. The mesh pores maintained an open state. The surface of the hydrogel coating is constructed with a large number of papillae with a size of 80–500 nm (Figure 14a,b). In water, oil droplets on the hydrogel-coated mesh have an OCA of 155.3° ± 1.8° (Figure 14c), so the mesh exhibits superoleophobic property underwater in the multiphase system. The oil droplets can roll off on the mesh easily (Figure 14d). The capability of the hydrogel-coated mesh in oil/water separation was investigated by fixing the mesh between two tubes and adding the mixture into the designed separation system (Figure 14e). The mesh was wetted by water in advance. As the mixture of crude oil and water was poured onto the superwetting mesh, only the water quickly permeated through the mesh while the oil was intercepted and could not pass through the mesh. No oil could be seen in the separated water, demonstrating that the crude oil and water mixture was effectively separated (Figure 14f). The separation efficiency was as high as 99%. Figure 14g depicts the mechanism of separating oil/water mixture via underwater superoleophobic porous mesh. The superhydrophilicity of the mesh allows the water phase to pass through the mesh freely. On the contrary, the underwater superoleophobic mesh has a remarkable repellent effect on oils so that the oils are unable to penetrate the mesh and thus stay above the mesh. The mixture of oil and water is then separated into the oil part (above the separation membrane) and the water part (below the separation membrane).

**Figure 15:**
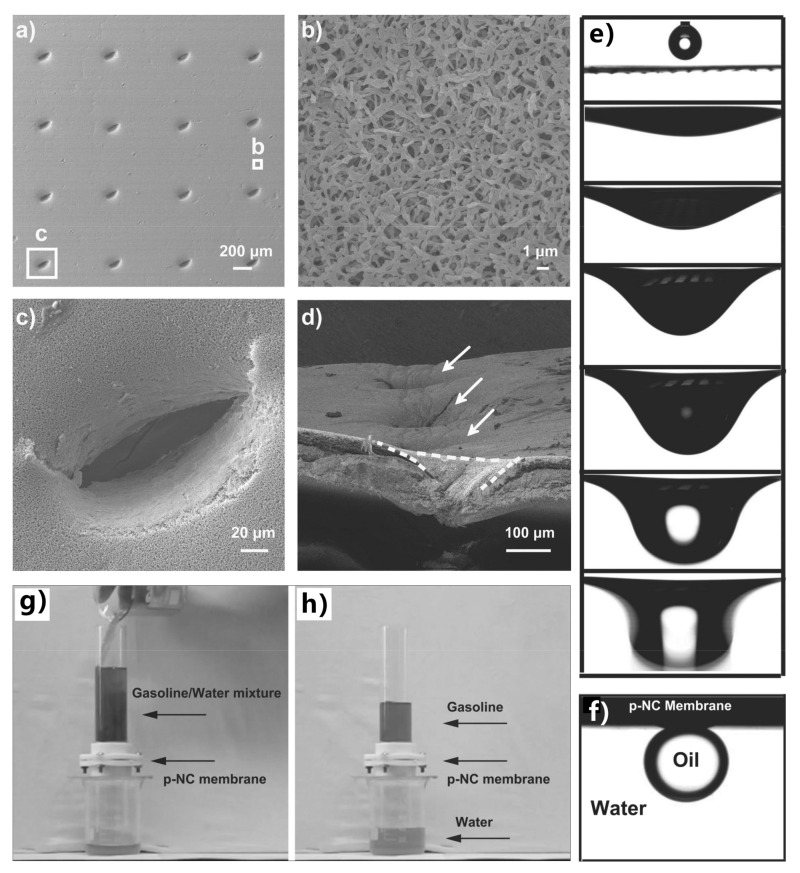
Achievement of the oil/water separation based on a dual-scaled porous nitrocellulose membrane. (**a**) SEM image of the dual-scaled porous nitrocellulose membrane. (**b**) Morphology of the inherent pore structure of the nitrocellulose substrate composed of interconnected nanofibers. (**c**) Top-viewed and (**d**) cross-sectional microstructure of the perforated hole on the nitrocellulose membrane. (**e**) Process of water droplets wetting and permeating through the porous nitrocellulose membrane. (**f**) A drop of light oil on the membrane underwater. (**g**) Pouring the mixture of gasoline and water into the separation device composed of the perforated membrane. (**h**) After separation. Reprinted with permission from ref. [262]. Copyright 2013, WILEY-VCH Verlag GmbH & Co. KgaA.

Gao et al. used a dual-scaled porous nitrocellulose membrane to achieve highly efficient oil/water separation [262]. The nitrocellulose substrate is made up of nanofibers. The nanofibers interconnect so that pores form between the nanofibers (Figure 15b). The average size of the pores is 450 nm. A series of bigger pores were produced on the nitrocellulose membrane by the simple perforating method using a conical-tipped needle (Figure 15a). The perforated holes possess olivary top morphology (Figure 15c) and a concave cross-sectional profile (Figure 15d). When water droplets were dripped onto such a membrane, they spread over quickly and permeated through the membrane freely because of the superhydrophilicity of the membrane and the perforated microholes (Figure 15e). After water immersion, the perforated membrane exhibit underwater superoleophobicity to various oils with an OCA of ~156.4° ± 3.2° (Figure 15f). When a mixture of gasoline and water was released on the perforated membrane sandwiched between two tubes previously (Figure 15g), water permeated through the membrane quickly while gasoline was repelled and maintained above the underwater superoleophobic membrane (Figure 15h). Thus, the mixture of gasoline and water was quickly separated. The membrane presents high efficiency (above 99%) for separating water and various oils.

**Figure 16:**
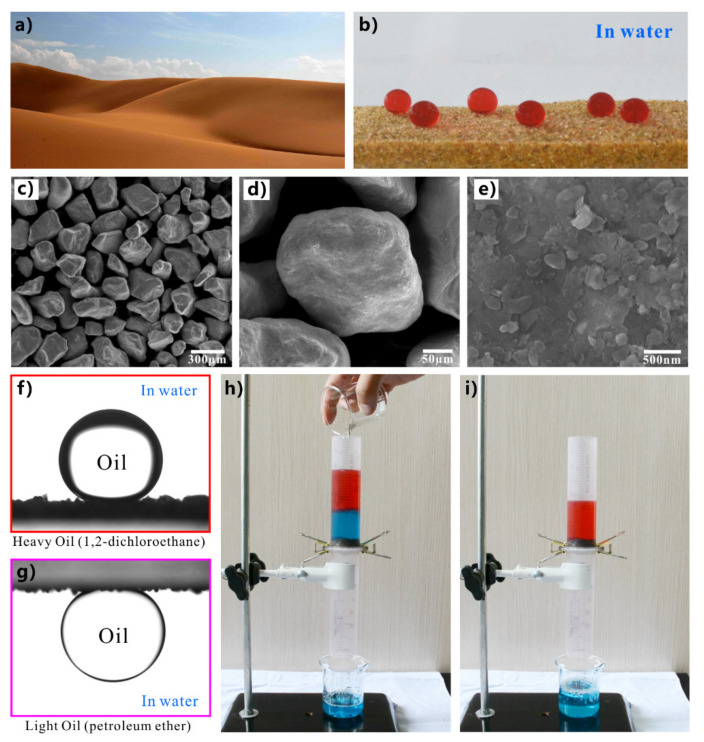
Simply separating the oil/water mixture by prewetted sand layer. (**a**) Desert. (**b**) Drops of heavy oil on the sand in water. (**c**) SEM image of a sand layer composed of a large number of sand particles. (**d**) Morphology of a sand grain. (**e**) Surface microstructure of a sand grain. (**f**) Heavy and (**g**) light oil droplets on the sand layer underwater. (**h**,**i**) Separating the oil/water mixture by using the prewetted sand layer: (**h**) pouring the mixture of water (blue color) and oil (petroleum ether, red color) onto the sand layer and (**i**) after separation. Reprinted with permission from ref. [263]. Copyright 2016, WILEY-VCH Verlag GmbH & Co. KgaA.

Most of the earth is covered with vast deserts stretching thousands of miles (Figure 16a). The sand particles, which make up the whole desert, are tiny, with a diameter of only ~200 µm (Figure 16c). The surface of sand grains is decorated with microscale rough texture and abundant fine nanoscale particles and debris (Figure 16d,e). A large space exists between the sand particles for a layer of sand (Figure 16c). Yong et al. found the underwater quasi-superoleophobicity of the superhydrophilic sand layer in water (Figure 16b) [263]. Both heavy oil and light oil droplets could take the shape of a round bead on the sand layer underwater (Figure 16f,g). The measured OCA is 148.5° ± 2.5° to 1,2-dichloroethane (heavy oil) droplet and 149.5° ± 2° to petroleum ether (light oil) droplet, respectively. Oil droplets could roll off the sand surface easily. These results indicate that the sand surface is quasi-superoleophobic in water. The underwater quasi-superoleophobicity enables the sand layer to separate the oil/water mixture. A layer of sand with a thickness of 1 cm was supported by a piece of cloth in a plastic tube and was prewetted with a little water. As the mixture of water and oil (petroleum ether) was fed into the designed separation device, only water permeated through the sand. In contrast, the oil always stayed above the sand layer during the entire separation process (Figure 16h). The mixture was finally separated using the sand layer as the separation membrane (Figure 16i). Since the sand is taken directly from the desert without any treatment, this separation process is very low cost and very green.

Zeolite is rich in unique pore structure and has the feature of mechanical, thermal, and chemical stability. Wen et al. coated pure-silica zeolite crystals on the wire of a metal mesh by a growing method [264]. The superhydrophilicity and underwater superoleophobicity of the zeolite coating enabled the high-efficiency separation of various oils by using the as-prepared mesh. The passage of water through the mesh was allowed while that of oil was prohibited. In addition, the zeolite film could be resistant to corrosion of various corrosive liquids; thereby, such a mesh could separate a variety of the mixtures of water and oils even under a harsh environment. Zhang et al. immersed a copper mesh into the solution of alkaline ammonium persulfate, resulting in the formation of inorganic nanowires on the mesh after chemical oxidation [265]. The coating of the nanowires led to superhydrophilicity and underwater superoleophobicity for the metal mesh. The mixture of layered oil and water and the oil/water emulsion could be effectively separated by the as-prepared underwater superoleophobic mesh. This mesh could even separate high-viscosity oils. Song et al. dipped a copper mesh in the cement paste and obtained a cement-coated mesh [266]. The cement-coated mesh showed superhydrophilicity and underwater superoleophobicity as the hydroxyl groups, and the micro/nanostructures of the cement coating were integrated into the mesh surface. When the cement-coated mesh was used as a separation membrane, even the mixtures of oils and harsh aqueous solutions (e.g., alkaline solution, salt solution, and hot water) could be separated. In addition, the separation process could be efficiently repeated at least 30 times. Such a separation material was prepared cost-effectively and environment-friendly, without using acid, alkali, and organic reagents. Dai et al. deposited graphene oxide (GO) and CaCO_3_ on a stainless-steel mesh through the layer-by-layer self-assembly method [267]. The nacre-like underwater superoleophobic microstructures of graphene oxide-calcium carbonate (GO-CaCO_3_) formed onto the wire surface of the mesh. When a mixture of cyclohexane and water was in contact with the water-wetted hybrid mesh, the water in the mixture freely passed through the mesh, and the underwater superoleophobicity made the cyclohexane be blocked above the hybrid mesh. The water and cyclohexane were well separated, with the separation efficiency higher than 99%.

Chen et al. deposited CaCO_3_ nanoparticles on a polypropylene microfiltration membrane that was pre-grafted with polyacrylic acid [268]. The rigid mineral-coating could trap water in its pore structure. In an aqueous environment, a hydrated layer formed on the membrane, resulting in the underwater superoleophobicity of the hybrid membrane. Such a hybrid membrane could separate both the immiscible oil/water mixture and the emulsion of oil in water. The separation efficiency was higher than 99%, the water flux was higher than 2000 L m^−2^ h^−1^, and the breakthrough pressure to oil was larger than 140 kPa in the process of oil/water separation. Xue et al. peeled complete polyvinylidene fluoride (PVDF) film with hierarchical rough microstructures from the surface of non-woven fabric through the phase-conversion method [269]. The formation of the rough multi-level structure made the film surface have suitable underwater superoleophobicity and underoil superhydrophobicity. The special wettability allowed the film to separate the mixture of water and oil and the oil-in-water and water-in-oil emulsions.

Yong et al. drilled microholes on a metal sheet by a mechanical process and further prepared ripple-like nanostructure on the porous metal sheet via femtosecond laser processing [270]. The structured porous metal sheet has great underwater superoleophobicity with an OCA of 164° in water. The oil/water mixtures were easily separated by the laser-structured porous sheet because of the microhole structure and underwater superoleophobicity of the sheet. Oil could not pass through the sheet because the superoleophobicity of the sheet provided the repellence to oil, but the water quickly permeated through the porous sheet. Li et al. prepared a porous membrane through 3D printing technology [271]. The printed ink is composed of poly (viny1 alcohol), cellulose acetate, and silica nanoparticles. The printed membrane is underwater superoleophobic and has a remarkable ability to separate the mixtures of water and various oils (e.g., n-hexane, vegetable oil, diesel oil, and lubricating oil). Compared with the traditional metal mesh-based separation materials, the printed membrane had superior mechanical stability. The separation efficiency was maintained at ~99.0% even when the membrane was subjected to ultrasonic treatment for 30 min or bent 100 times. Zhang et al. fabricated a chitosan/nanofibrillated cellulose aerogel by freeze-drying method [272]. The nanofibrillated cellulose was incorporated into the chitosan matrix. Together with the inherent hydrophilicity of chitosan, the hybrid aerogel was superoleophobic in water. The underwater superoleophobicity was stilled exhibited even though the aerogel was immersed in high-salinity seawater for about a month. The features of underwater superoleophobicity, high porosity, and salt tolerance enabled the aerogel to efficiently separate various oils (such as kerosene, soybean oil, n-hexane, toluene, and crude oil) from seawater mixtures.

### 4.3. Superhydrophobic 3D Porous Materials for Oil Absorption

Using the superhydrophobic or underwater superoleophobic porous mesh/membranes, the oil/water separation is achieved by filtering. However, the filtering method is not the best choice in special cases, such as a small amount of oil leaking on or below the water surface. The transportation of the mixture of oil and a large volume of water onto the separation membrane will cost a great deal of workforce and resources. The oil pollution can be just precisely removed by an oil absorption material. Apart from the superwetting porous filtration film, the 3D porous bulk materials with superhydrophobicity and superoleophilicity can also be used to separate the mixture of water and oil based on an absorption manner [273,274,275,276,277,278,279,280,281,282]. This kind of material can absorb oil directly from the water to achieve the function of removing or collecting oil.

**Figure 17:**
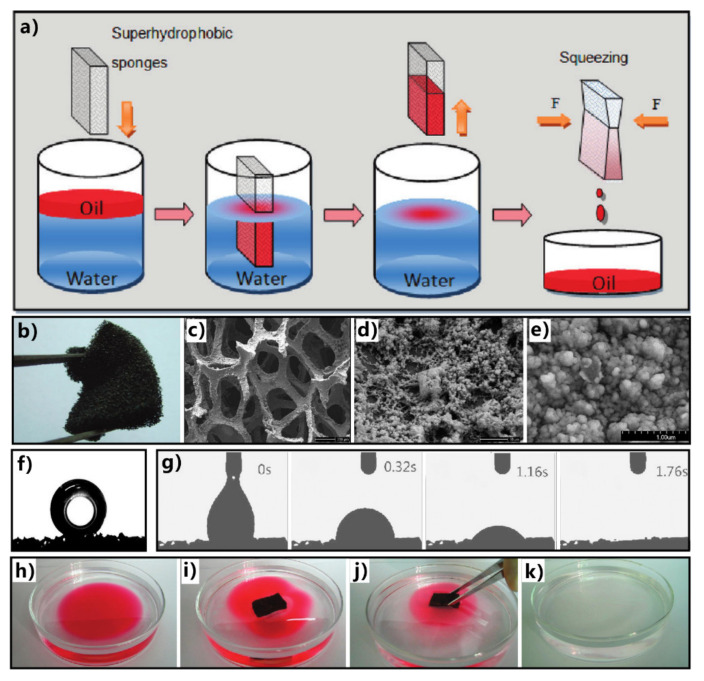
Absorption-based separation of oil from water by a superhydrophobic/superoleophilic sponge. (**a**) Schematic of the process of removing oils from water within a cycle of oil absorption, transportation, and release. (**b**) Photo of the superhydrophobic and superoleophilic porous PU sponge. (**c**) SEM image of the as-prepared sponge. (**d**,**e**) Surface microstructure of the sponge skeleton. (**f**) Water droplet on the structured sponge. (**g**) Oil being absorbed by the as-prepared sponge. (**h**–**k**) Using a superhydrophobic sponge to selectively absorbing oil (red color) from the water. Reprinted with permission from ref. [283]. Copyright 2011, American Chemical Society.

Figure 17a shows the mechanism and process of removing oils from water by superhydrophobic porous 3D materials, taking a superhydrophobic and superoleophilic sponge as an example [283]. When the sponge is moved to contact with the oil on the water surface or underwater, superhydrophobicity enables the sponge surface to repel water. Thus, water cannot enter such a porous 3D material, while the sponge can quickly absorb oil due to the superoleophilicity. As the oil is completely absorbed, the sponge is taken out of the water. The oil is also removed from the water with the sponge. The absorbed oils can be easily released to the target location by squeezing treatment and be collected for re-use. Both the oil on the water surface and the oil under the water can be completely removed from water by the superhydrophobic and superoleophilic absorption materials through the cycle of oil absorption, transferring, and releasing. When the compressed sponge was recovered, it could be used again to separate oil from water.

Zhu et al. used a superhydrophobic and superoleophilic sponge to successfully remove or collect oils from the water surface [283]. The porous polyurethane (PU) sponge was adopted as the substrate. The superhydrophobic microstructures were prepared on the skeleton of the sponge through a simple solution-immersion process. The sponge became dark brown but maintained its porous appearance and elastic property (Figure 17b). The sponge can easily deform under squeezing. The pore size of the interconnected sponge framework ranges from 200 to 450 μm (Figure 17c). Abundant nanoparticles with a grain size of 100–200 nm cover the sponge’s skeleton (Figure 17d,e). The synergy of the low-surface-energy chemical modification and the hierarchical microstructure results in the superhydrophobicity of the as-prepared sponge (Figure 17f). Besides the water-repellent property, the as-prepared sponge also displays superoleophilicity and can absorb oil quickly (Figure 17g). The interconnected porous framework provides excellent space for oil adsorption. When the sponge was placed on the surface of water contaminated with oil (Figure 17h), it quickly picked up the oil on the water while water could not be absorbed by such a superhydrophobic sponge (Figure 17i,j). As the sponge was pulled out of the water, the oil was removed entirely with the superwetting sponge (Figure 17k). The oil could be squeezed out from the sponge and be collected. The sponge could selectively absorb a range of oils, and the absorption capacity was 13 times higher than the mass of the sponge. The result demonstrates that the superhydrophobic sponge has high efficiency and high selectivity in the application of oil/water separation.

**Figure 18:**
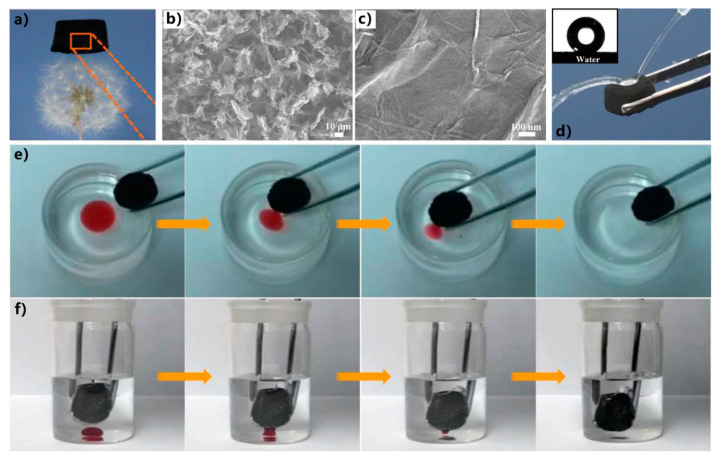
Absorption and removal of oil from water through a superhydrophobic graphene aerogel. (**a**) Photo of a piece of cylindrical aerogel on the top of a dandelion. (**b**,**c**) Morphology of the graphene aerogel. (**d**) Water flow bouncing off the surface of the aerogel. Inset: water droplet on the superhydrophobic graphene aerogel. (**e**) Selectively adsorbing oil (toluene) floating on the water surface by the as-prepared graphene aerogel. (**f**) Selectively adsorbing heavy oil (chloroform) underwater by the as-prepared graphene aerogel. Reprinted with permission from ref. [284]. Copyright 2018, American Chemical Society.

Aerogels have the characteristics of ultralight, excellent mechanical strength, high porosity, and extraordinary adsorption capacity. Yang et al. prepared an ultralight superhydrophobic graphene aerogel by a vapor-liquid deposition strategy [284]. The monolayer of 1H,1H,2H,2H-perfluorooctyltriethoxysilane was finally modified on the dry aerogel via the chemical vapor deposition. The fluorinated graphene aerogel is ultralight, whose density is only 4.8 ± 0.3 mg/cm^3^. When a piece of cylindrical aerogel (with a volume of 2.4 cm^3^) was placed on the top of a dandelion, it could stably stand without destroying the fluffs of the dandelion (Figure 18a). As shown in Figure 18b,c, a 3D interconnected porous network structure characterizes the graphene aerogel. The size of the pores ranges from a submicrometer to 10 μm. Many nanoscale granules are also randomly distributed on the graphene surface. The as-prepared graphene aerogel shows superoleophilicity (inset of Figure 18d). A flow of water can easily bounce off the surface of the aerogel (Figure 18d). By contrast, oil droplets can be completely absorbed by the graphene aerogel. The superhydrophobic aerogel was successfully used to selectively adsorb the floating oils on the water and the heavy oils underwater. When a piece of the aerogel was moved to touch the toluene oil on the water, the oil layer around the aerogel was adsorbed by the aerogel within a few seconds (Figure 18e). Likewise, the chloroform sinking to the bottom of water could also be immediately sucked into the aerogel as the aerogel was dipped in water and made contact with the droplet of chloroform (Figure 18f).

Pan et al. dipped a PU foam into a solution containing dopamine and Fe_3_O_4_ nanoparticles [285]. After being removed to dry, a superhydrophobic and superoleophilic PU foam modified with dopamine-coated Fe_3_O_4_ nanoparticles was prepared by the self-assembly method. Such a foam was successfully used to absorb the oils from the water surface and achieve the oil/water separation. Mi et al. prepared a superhydrophobic polypropylene (PP)/PTFE composite foam based on the twin-screw extrusion and supercritical carbon dioxide foaming [286]. PTFE particles on the scale of microns and nanometers were melt blended with PP by the twin-screw extruding method. Then, the supercritical CO_2_ was adopted to foam the composite material. As a result, a special foam with a highly porous structure was obtained. The web-like PTFE nanofibers and PTFE nanoparticles are evenly covered on the microsized PP pores. The superhydrophobic foam can absorb not only heavy oil but also light oil from water. For example, the hexane atop water and chloroform underwater could be selectively absorbed by the superhydrophobic foam.

Huang et al. developed a simple one-pot route to fabricate various superhydrophobic composite materials [287]. A homogeneous epoxy mixture was obtained by dissolving the epoxy resin and polyamide in the hydrophobic silica sol solution under magnetic stirring. Various materials can be adopted as the substrate to prepare superhydrophobic composite materials. For example, when a latex sponge was dipped into the mixture, the epoxy resin was cured with an amine at a reaction temperature of 45 °C to produce epoxy resin microspheres. The microspheres could adhere to the porous framework of latex. After soaking for a day, the latex was removed, washed with ethanol, and then dried in the air. The latex composite material showed robust superhydrophobicity, which allowed the 3D porous latex to selectively absorb oils from the water surface. Qiu et al. used the typical one-step ultrasonic dip-coating process to modify a porous PU sponge [288]. The sponge was dipped into the solution composed of the superhydrophobic sepiolite powder, which was previously modified by octadecyl trimethylammonium bromide and octadecyltrichlorosilane. A layer of rough micro/nanostructures was generated on the skeleton surface of the sponge. The generated microstructures and the low surface energy of the octadecyltrichlorosilane endowed the sponge with superhydrophobicity. The resultant porous sponge became an excellent adsorbent material due to its superhydrophobic and superoleophilic properties, which could selectively adsorb oil in water while completely repelling water.

He et al. prepared a superelastic and superhydrophobic aerogel through vacuum infiltration and freeze-drying [289]. The 3D self-assembled bacterial cellulose was used as the skeleton, and the methyltriethoxysilane-derived silica aerogel was used as filler. The aerogel exhibits superhydrophobicity with a WCA of 152° and superoleophilicity, endowing the aerogel with selective oil-absorbing capability. The aerogel could withstand up to 80% of compressive strain and return to its original shape after stress release, allowing the absorbed oil to be easily retrieved by mechanical compression.

Yu et al. proposed a strategy to realize the rapid adsorption of high-viscosity crude oil by using a hydrophobic/oleophilic sponge coated with GO [290]. Voltage was applied to provide Joule heat for the graphene-coated sponge. The generated Joule heat can significantly improve the adsorption rate of sponge to crude oil because the viscosity of the crude oil on the sponge decreases as the temperature of the sponge increases. The research provides a new strategy for efficient cleanup and recovery of high-viscosity oil spills, a global problem. Wu et al. coated carbon nanotubes and polypyrrole on a commercial melamine sponge [291]. The sponge has stable superhydrophobicity and oleophilicity. The introduced carbon nanotubes/polypyrrole coating can convert light and electricity into heat, causing the temperature of the sponge to rise under solar irradiation and applied voltage. The heat generated on the sponge surface reduces the viscosity of crude oil and dramatically promotes the absorption process of crude oil to the sponge. For example, the as-prepared sponge could reach a high temperature of 118.6 °C at 1 sun illumination (1.0 kW/m^2^) and 8 V voltage. In this case, the penetration time of oil droplets decreased by 93.5% compared to an unheated sponge. By connecting to a peristaltic pump, the sponge could continuously clean oil from the surface of the water.

**Figure 19:**
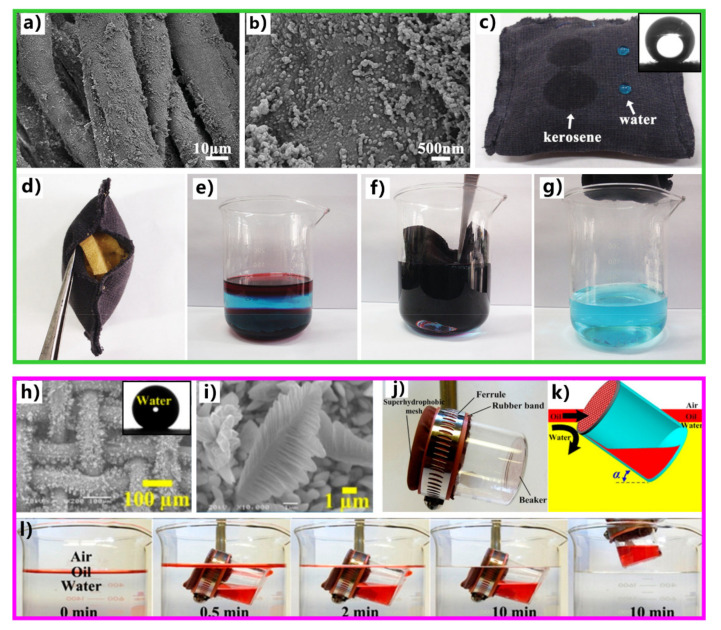
Transforming superhydrophobic 2D porous mesh/membrane into 3D oil-absorption materials. (**a**–**g**) Superhydrophobic and superoleophilic oil-absorption cloth bag: (**a**) surface microstructure of the nanoparticles-coated fabric, (**b**) microstructure on the fabric surface, (**c**) drops of water and oil (kerosene) on the cloth bag (inset: water droplet on the superhydrophobic fabric), (**d**) Photo of the superhydrophobic cloth bag prepared by sewing the as-prepared fabrics to a bag and filling with an original sponge, (**e**) oil/water mixture before separation, (**f**) selectively absorbing oils from water by using the superhydrophobic cloth bag, and (**g**) after removing oils from the mixture. Oil was dyed with red color, and water was dyed with blue color. (**h**–**l**) Superhydrophobic and superoleophilic oil-absorption container: (**h**) morphology of the textured stainless-steel mesh (inset: water droplet on the as-prepared mesh), (**i**) microstructure on the mesh surface, (**j**) the artificial oil-absorption container prepared by installing the superhydrophobic metal mesh on the mouth of the glass beaker, (**k**) schematic of absorption and collection of the oil floating on the water, and (**l**) removing oil floating on the water by the oil collection container. Reprinted with permission from ref. [292]. Copyright 2016, WILEY-VCH Verlag GmbH & Co. KgaA. Reprinted with permission from ref. [293]. Copyright 2014, American Chemical Society.

The absorption manner is not confined to 3D materials. The 2D porous mesh/membrane can also be transformed into a 3D bulk material with a superhydrophobic outer surface. For instance, Li et al. fabricated a superhydrophobic cloth bag [292]. The SiO_2_ nanoparticles functionalized by octadecyltrimethoxysilane were grafted onto the surface of the fabric by simple sonochemistry irradiation (Figure 19a,b). The water droplet on the as-prepared fabric has a WCA of 158° ± 1°, and the kerosene droplet on the fabric has an OCA of 0°, so the fabric is simultaneously superhydrophobic and superoleophilic (Figure 19c). In order to prepare the superhydrophobic cloth bag, the superwetting fabrics were sewn into the cloth bag, and the original sponge with high adsorption capacity was filled into the bag (Figure 19d). The outer superhydrophobic cotton cloth is used as a filter membrane that only allows oil to selectively penetrate, and the oil is finally absorbed by the filling sponge. When the bag was inserted in a mixture of water, kerosene (on the water), and chloroform (underwater), the oils were quickly absorbed by the bag because of its superoleophilicity (Figure 19e,f). By contrast, the water was unable to enter into the superhydrophobic bag (Figure 19g). The bag structure significantly improves the absorption volume of the superhydrophobic separation material. With the introduction of a negative pressure system, the whole system could adsorb and remove oils from the oil/water mixture at high speed, continuously and efficiently. Such a separation bag combines the high selectivity of the superwetting fabric with the high absorption capacity of the porous bulk sponge. Similarly, Song et al. prepared an oil-absorption container based on a plane superhydrophobic mesh [293]. Superhydrophobic and superoleophilic leaf-like microstructures were firstly prepared on the surface of the stainless-steel mesh (Figure 19h,i). Then, the metal mesh was installed on the mouth of the glass beaker, as shown in Figure 19j. To remove oils (e.g., hexadecane) floating on the water surface, the container was slightly tilted (e.g., at 22°) and partly immersed in the floating oil/water mixture, ensuring that part of the mesh was exposed to air and the other part was submerged in liquid (Figure 19k). The floating oil film could pass through the mesh and enter into the container. In contrast, the entrance of water into the collection container was refused by the superhydrophobicity of the mesh (Figure 19l). In this way, the oil floating on water was removed and collected by such a container capped with the superhydrophobic mesh.

Extreme wettability and porous microstructure are necessary to achieve effective and repeatable oil/water separation. The reported superwetting materials and the separating ways used in separation are not limited to the abovementioned three types. The mixtures of water and oil are often diverse, including the cases of oils on the water surface, oils underwater, water-in-oil emulsion, oil-in-water emulsion, and so on. To perform efficient oil/water separation, the separating materials should give play to their respective characteristics in structure and wettability, depending on the different types and properties of the oil/water mixtures.

A growing number of examples witness the success of superwettability in oil/water separation applications [1,4,10,17,19,20]. The separation of water and oil is achieved based on the property that water and oil have different wetting behaviors on the superwetting porous separation materials. The oil/water mixture is just the tip of the iceberg, and a large number of other mixtures also need to be separated. For example, removing and collecting the various liquid polymer pollutants leaked into the water are difficult as the liquid polymers readily adhere to any solid material. In addition to liquid/liquid separation, in some cases, the gas in liquid also needs to be removed or collected. It is of great significance to extend the wettability-based oil/water separation process to other types of liquid/liquid mixtures and liquid/gas mixtures.

## 5. Polymer/Water Separation Based on the Underwater Superpolymphobic Materials

Polymers are broadly applied in the chemical industry, food package, energy development, lifestyle products, pharmaceuticals, building, agriculture, etc. Some polymers are liquid-state besides the common solid-state. With the widespread applications of liquid polymers, the leakage of liquid polymers into the water results in big waste and even various polymer pollutants in water [294,295,296,297]. Traditionally, three representative methods are used to remove polymer pollutants from wastewater: (a) adsorption using solid adsorbents such as activated carbon, (b) detoxication treatments such as chemical oxidation and photodegradation, and (c) solidifying and separating from water by coagulation, flocculation, and precipitation. However, high-viscosity and low-fluidity properties make the liquid polymers easy to adhere to any solid material. So, the liquid polymers are difficult to remove from water effectively and on a large scale using these standard methods. Inspired by the process of oil/water separation, the superwettability to the liquid polymer can potentially achieve the separation of water and polymer.

In 2019, the phenomenon of a surface greatly repelling liquid polymers underwater was reported by Yong and co-authors [298,299,300,301]. In water, a tiny droplet of liquid polymer on the underwater superpolymphobic surface has a polymer CA (PCA) larger than 150°. Such superwettability is defined as underwater superpolymphobicity. The underwater superpolymphobic surfaces show remarkable repellence to liquid polymers in water. The repellence of underwater superpolymphobic materials to liquid polymers provides a possibility to separate polymer from water to alleviate the waste and environmental pollutions caused by liquid polymers.

### 5.1. Underwater Superpolymphobicity

**Figure 20:**
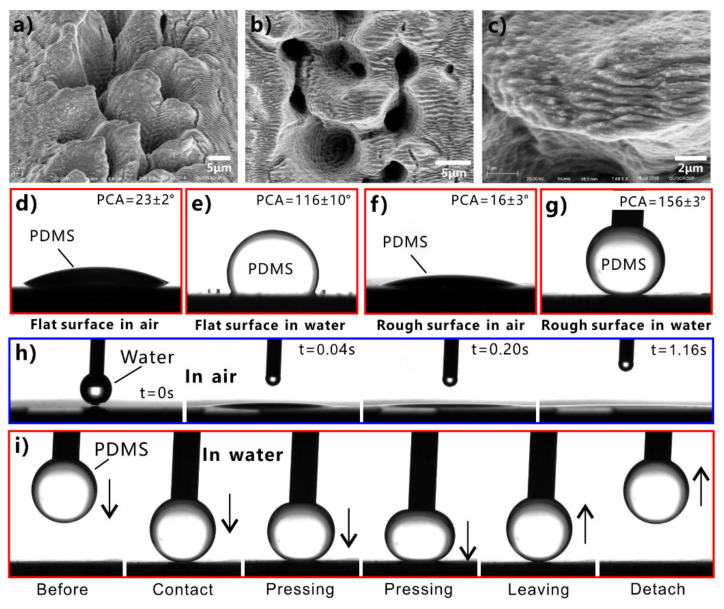
Underwater superpolymphobicity of the stainless steel with hierarchical surface microstructures. (**a**–**c**) SEM images of surface microstructures on the stainless-steel surface. (**d**) A droplet of liquid polymer on the original stainless steel in air. (**e**) A droplet of liquid polymer on the original stainless steel in water. (**f**) A droplet of liquid polymer on the structured stainless-steel surface in air. (**g**) A droplet of liquid polymer on the structured stainless-steel surface in water. (**h**) A water droplet wetting the structured surface. (**i**) Moving an underwater liquid polymer droplet to contact and then leave the structured stainless-steel surface. Reprinted from ref. [299].

Similar to typical superhydrophobic or superoleophobic states, the formation of surface microstructures is also crucial for making a substrate repel liquid polymers. Yong et al. first achieved underwater superpolymphobicity by creating proper microstructures on a hydrophilic surface [299]. Femtosecond laser processing was used to directly prepare microstructures on the surface of stainless steel. The surface of the structured stainless steel is characterized by mountain-shaped microstructures. The micromountains have a diameter of ~5.2–11.7 μm and a height of ~27.5 μm (Figure 20a). The micromountains are surrounded by deep microholes (Figure 20b). The diameter of the microholes is ~2.5–9.6 μm, and their depth can reach up to 19.4 μm. Periodic ripple-like nanostructures are also formed on the surface of the micromountains (Figure 20c). The nanoripples are about 352 nm in width. The original stainless steel is hydrophilic, with an intrinsic WCA of 79° ± 2°. The untreated stainless steel is also inherently polymphilic in the air as the PCA of a liquid polymer droplet on its surface is only 23° ± 2° (Figure 20d). When the sample was submerged in water, the liquid polymer droplet on the smooth stainless steel had a PCA of 116° ± 10° (Figure 20e), indicating that the stainless-steel substrate shows polymphobicity underwater. The polymer droplet was firmly stuck on the stainless-steel surface even though the substrate was placed vertically. The structured stainless-steel surface becomes superhydrophilic. Water droplets could quickly spread out and wet the ablated area (Figure 20h). The PCA of the in-air polymer droplet decreases to 16° ± 3° (Figure 20f). Interestingly, a liquid polymer droplet can maintain spherical on the structured surface in water, with a PCA of 156° ± 3° (Figure 20g). The adhesion between the structured stainless steel and the underwater liquid polymer is ultralow (Figure 20i). The CA hysteresis (CAH) of the polymer on the sample surface is less than 4°. Therefore, the stainless-steel surface shows underwater superpolymphobicity after the formation of surface micro/nanostructures. The underwater superpolymphobic materials have remarkable repellence to liquid polymers in water.

**Figure 21:**
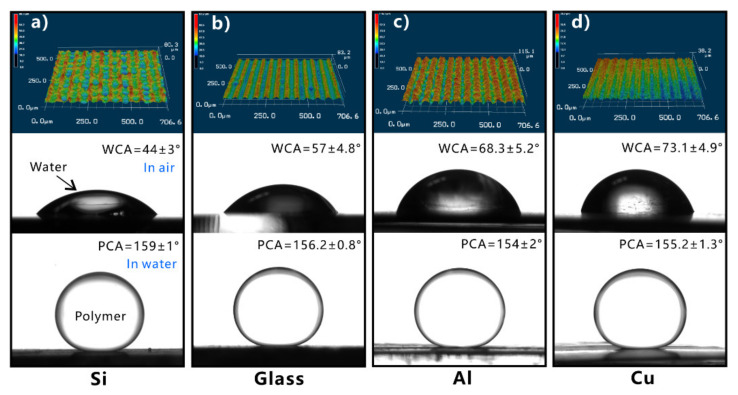
Underwater superpolymphobicity of the (**a**) silicon, (**b**) glass, (**c**) aluminum, and (**d**) copper surfaces after the preparation of surface microstructures. First line: Surface microstructures on the substrates. Second line: In-air water droplets on the original substrates without surface microstructures. Third line: A droplet of liquid polymer on the structured surfaces in water. Reprinted from ref. [300].

The underwater superpolymphobicity can be achieved on a wide range of other hydrophilic substrates by creating a proper surface microstructure, similar to the stainless steel. As shown in Figure 21, silicon, glass, aluminum, and copper are also intrinsically hydrophilic materials. Water droplets on the untreated smooth surfaces of those materials have a WCA of 44° ± 3° (silicon), 57° ± 4.8° (glass), 68.3° ± 5.2° (aluminum), and 73.1° ± 4.9° (copper), respectively [300]. When those surfaces are ablated by laser, micro/nanoscale structures are produced on the sample surfaces. Liquid polymer droplets on the structured silicon, glass, aluminum, and copper surfaces have spherical shapes in a water medium. The PCAs of the droplets are 159° ± 1° (on the silicon, Figure 21a), 156.2° ± 0.8° (on the glass, Figure 21b), 154° ± 2° (on the aluminum, Figure 21c), and 155.2° ± 1.3° (on the copper, Figure 21d), respectively [300]. The liquid polymer droplets have difficulty adhering to these surfaces because the underwater superpolymphobicity allows those surfaces to significantly repel liquid polymer in water.

**Figure 22:**
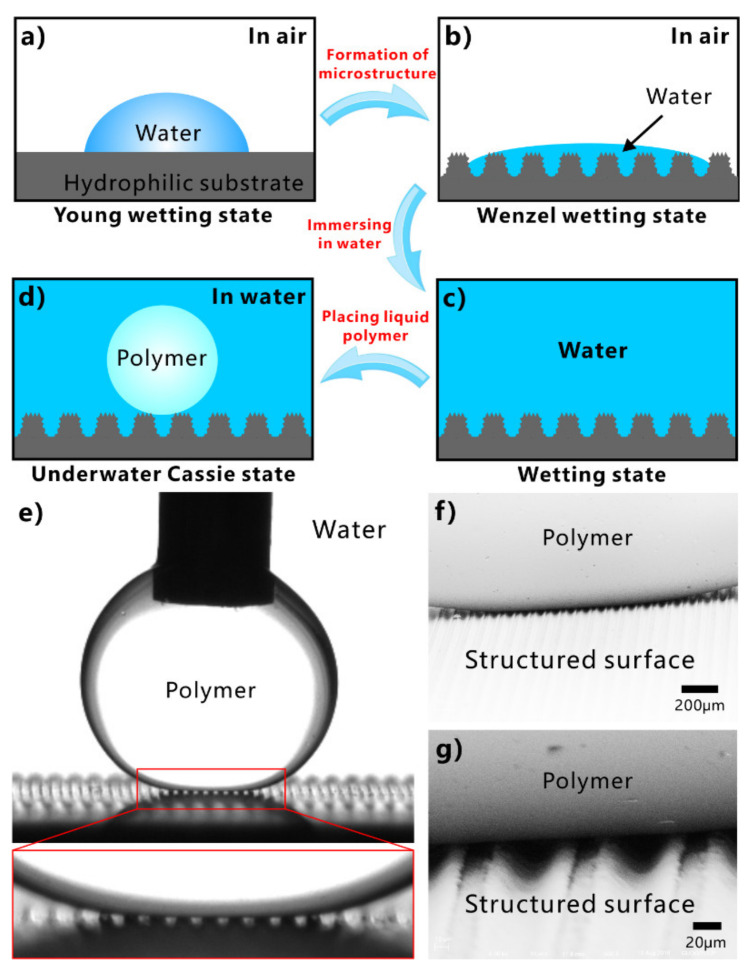
Formation mechanism of the underwater superpolymphobicity. (**a**) Water droplet on a smooth hydrophilic substrate. (**b**) Water fully wetting the structured surface. (**c**) The rough surface in a water medium. (**d**) Wetting state of a liquid polymer droplet on the rough surface in water. (**e**) Enlarged optical microscope image of the interface between the liquid polymer and the superpolymphobic microstructure in water. (**f**,**g**) SEM images of a droplet of liquid polymer (PDMS) on the sample surface after solidifying the polymer and removing the water environment. Reprinted from ref. [299].

Figure 22a–d describes the formation mechanism of the underwater superpolymphobicity [299]. The hydrophilicity of the hydrophilic substrate (Figure 22a) can be amplified to extrema state (e.g., superhydrophilicity) by the formation of surface microstructure that dramatically increases the surface area of the material [26,36,82,83]. The microstructure of a superhydrophilic surface can be thoroughly wetted by water, agreeing well with the Wenzel contact state (Figure 22b). Once the structured surface is submerged in water, all the spaces between the superhydrophilic surface microstructures will be filled with water medium (Figure 22c). When an underwater polymer droplet is further put on such a surface, a water film trapped in the surface microstructures forms underneath the polymer. Liquid polymer and water are insoluble and repel each other, so the trapped water cushion prevents the polymer from effectively contacting the substrate. The liquid polymer can only touch the tips of the surface microstructures (Figure 22d). The liquid polymer droplet on the solid surface is at the underwater Cassie contact state [26,36,82,83]. Such a contact state can be verified by experiment. Figure 22e shows the enlarged optical microscope image of the interface between the liquid polymer and the superpolymphobic microstructure in water [299]. The sequential white light spots between the polymer and the solid surface are the background light that freely passes through these regions, indicating that the white areas are filled with water. Figure 22f,g presents the actual situation of a droplet of liquid polymer on the sample surface after solidifying the polymer and removing the water environment [299]. The results demonstrate that the liquid polymer only touches the top of the surface microstructures of the underwater superpolymphobic materials. The contact area between the liquid polymer and the solid surface is significantly reduced by forming the surface microstructure, so the surface has underwater superpolymphobicity and strongly repels liquid polymers in water.

Although the formation mechanism of underwater superpolymphobicity is similar to that of underwater superoleophobicity, they have two different superwetting properties. Underwater superpolymphobic surfaces have special applications because they repel liquid polymers rather than oils. For example, underwater superpolymphobicity prevents polymers from sticking to substrates and can be used to manipulate liquid polymers. What is more, many liquid polymers can be converted from liquid to solid, allowing them to fix their shape. None of these phenomena or applications can be found in water or oil.

### 5.2. Polymer/Water Separation

**Figure 23:**
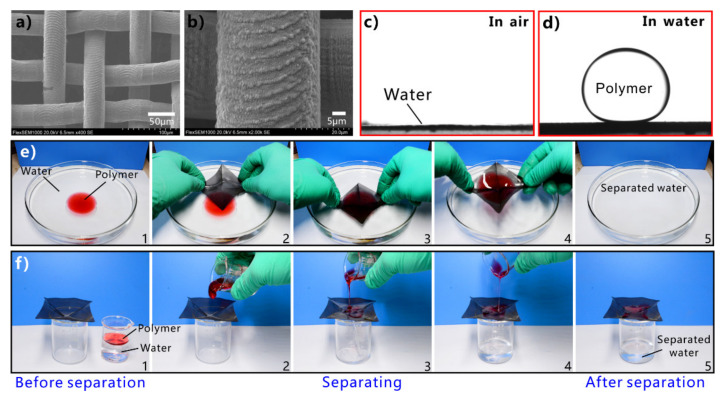
Separation of liquid polymer and water by using the underwater superpolymphobic mesh. (**a**,**b**) Surface microstructure of the laser-ablated stainless-steel mesh. (**c**) Water fully wetting the structured mesh. (**d**) A droplet of liquid polymer on the structured mesh in water. (**e**) Removing liquid polymer on the water through the collecting manner. (**f**) Separating the polymer/water mixture by the filtration manner. Reprinted with permission from ref. [302]. Copyright 2020, Elsevier.

The significant polymer repellence allows the underwater superpolymphobic materials to potentially be used to separate the mixtures of water and liquid polymers [15,298,302]. Yong et al. obtained underwater superpolymphobicity on a stainless-steel mesh via femtosecond laser treatment [302]. The hierarchical microstructure was uniformly formed on the mesh wire (Figure 23a,b). Microscale wave-like protuberances and nanoripples entirely cover the wire surface. In the air, when a water droplet was released onto the structured mesh, it would thoroughly wet the mesh (Figure 23c). The laser-ablated mesh shows superhydrophilicity in the air. With increasing the number of the dripped water droplets, the water finally permeated through the mesh. On the contrary, a liquid polymer droplet was repelled by the metal mesh in a water medium. The measured PCA is 156.4° ± 5.1° (Figure 23d) and the polymer SA (PSA) is 2.5° ± 0.5°, so the surface microstructures endow the mesh with excellent underwater superpolymphobicity. The superhydrophilicity and underwater superpolymphobicity enable the structured mesh to exhibit inverse wetting behaviors to water and polymers, respectively, making it possible for the mesh to separate various mixtures of water and liquid polymers. Figure 23e shows a strategy that uses underwater superpolymphobic mesh as a separation membrane to remove/collect the liquid polymers on the water. Before separation, the underwater superpolymphobic mesh was wetted by water to have the polymer-repellent ability. Then, the prewetted mesh was dipped into water and shifted to the position below the polymer layer. Like the fishing process, the mesh was finally pulled up. The superhydrophilicity of the mesh ensures that water freely passes through the metal mesh. On the contrary, the mesh repelled liquid polymer so that the polymer always stayed above the mesh because of the underwater superpolymphobicity. Thus, the liquid polymer was successfully removed from the water surface. Such a removal/collection process can remove a small volume of liquid polymer leaks in water.

The mixtures of liquid polymer and water can also be separated via filtration, as shown in Figure 23f. The mesh was also wetted by water in advance. As the polymer/water mixture was poured onto the prewetted mesh, only the water phase could permeate through the mesh and drip into the collecting beaker below. At the same time, the liquid polymer was intercepted by the structured mesh because of the underwater superpolymphobicity. The polymer in the mixture stayed above the mesh all the time. In this way, the mixture of polymer and water was separated, with a high separation efficiency of 99.0%. Liquid polymers cannot contaminate the underwater superpolymphobic mesh during the entire separation process due to its strong polymer repellence. Therefore, the separation process can recycle many times.

**Figure 24:**
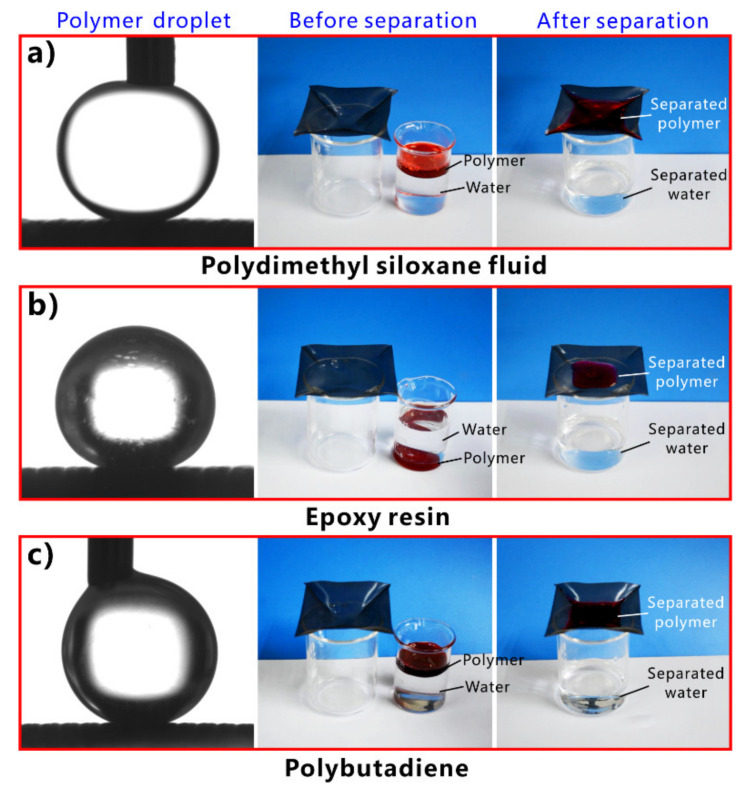
Separating different polymers from water by using an underwater superpolymphobic mesh: (**a**) polydimethylsiloxane fluid, (**b**) epoxy resin, and (**c**) polybutadiene. First column: an underwater droplet of liquid polymer on the structured mesh. Second column: before separation. Third column: after separation. Reprinted with permission from ref. [302]. Copyright 2020, Elsevier.

As the underwater superpolymphobic mesh exhibits strong repellence to a broad range of liquid polymers underwater, such a separation method can treat various water/polymer mixtures. For example, the liquid droplets of uncured PDMS (Figure 23d), polydimethylsiloxane fluid (Figure 24a), epoxy resin (Figure 24b), and polybutadiene (Figure 24c) have a spherical shape on the structured mesh in water [302]. All the droplets have a PCA larger than 150°. The mixtures of water and liquid PDMS (Figure 23f), polydimethylsiloxane fluid (Figure 24a), epoxy resin (Figure 24b), and polybutadiene (Figure 24c), respectively, could be effectively separated by the underwater superpolymphobic mesh. This polymer/water separation method has significant potential in polymer production and manufacturing, recovering waste polymer resources, and mitigating pollution from liquid polymer discharges.

The separation of polymer and water based on superwetting materials has a similar mechanism and process to the oil/water separation based on the underwater superoleophobic materials, while the application conditions of these two systems are completely different. Polymer/water mixtures are more difficult to separate than oil/water mixtures because liquid polymers generally have a higher viscosity, lower fluidity, and more complex composition than pure oil. Using underwater superpolymphobic materials to separate polymer/water mixtures can save polymer resources and prevent polymer-based environmental pollution.

The concept of underwater superpolymphobicity has been put forward in recent years, so the related research is still very limited. The underwater superpolymphobicity is a fascinating wetting property for a solid surface in manipulating polymers. The related applications of polymer/water separation will sprout like bamboo shoots after a spring rain.

## 6. Liquid/Gas Separation by Underwater Superaerophobic and Superaerophilic Materials

Bubbles often exist in aqueous solutions and are inevitable in the environment, industrial processes, agriculture, energy, etc. [303,304,305,306,307,308]. Sometimes, the bubbles in the liquid will cause various troubles. For example, bubbles usually increase the fluid resistance of a liquid in a microfluidic system. Even worse, they can block the microchannels. There is a risk of life and health of the patients if the air bubbles are injected into the blood vessel of a human in the process of infusion. When the gas bubbles produced in an electrochemical reaction are attached to the surface of the electrode, the efficiency of the chemical reaction will be significantly reduced because the adhered bubbles inhibit the effective contact between the electrodes and the electrolyte [309,310,311,312,313]. Many toxic and polluting gases are dissolved in industrial wastewater and are eventually released into the air environment with wastewater discharge, resulting in severe pollution to the environment. The separation of gas and liquids is a possible way to solve the bubbles-induced problems.

Like the wettability of water and oil, the wetting behavior of underwater bubbles on a solid surface is also worth studying. The bubble wettability has two extreme states. The surface is underwater superaerophobic when the bubble has a bubble CA (BCA) larger than 150° on the material surface and is underwater superaerophilic when the BCA of the bubble is less than 10° in a water medium. The materials with superwettability to bubbles can be used to manipulate bubbles in the water. Controlling the bubble wettability of a solid substrate has a broad application prospect, which is helpful to make rational use of bubbles and avoid the damage caused by bubbles in the liquid.

### 6.1. Underwater Superaerophobicity and Superaerophilicity

**Figure 25:**
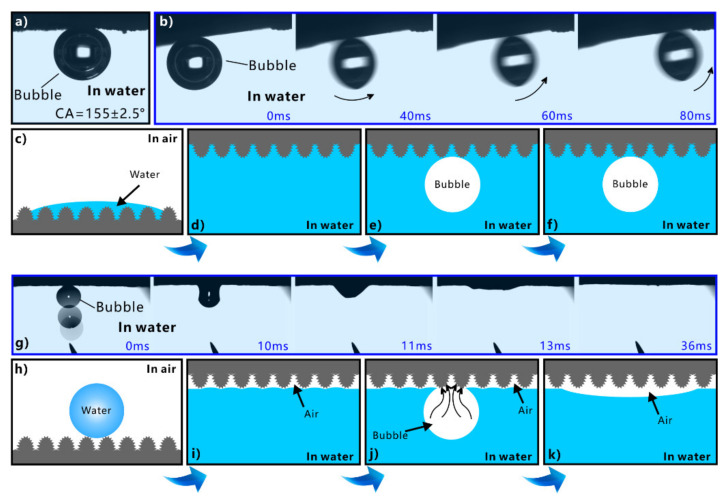
Superaerophobicity of fish scales and superaerophilicity of lotus leaves in water. (**a**) An air bubble on the surface of a fish scale underwater. (**b**) Bubble rolling away on the fish scale in water. (**c**–**f**) Schematic formation mechanism of the underwater superaerophobicity of fish scales: (**c**) water wetting the surface microstructures of fish scale in air, (**d**) immersion of the fish scale in water, (**e**) underwater bubble just contacting with the fish scale, and (**f**) after some time. (**g**) Process of a bubble touching the surface of a lotus leaf underwater. (**h**–**k**) Formation mechanism of the underwater superaerophilicity of lotus leaf: (**h**) water being repelled by the superhydrophobic microstructures of leaf surface, (**i**) lotus leaf in water, (**j**) releasing a bubble onto the lotus leaf in water, and (**k**) after some time. Reprinted with permission from ref. [46]. Copyright 2017, American Chemical Society.

Fish can swim freely in water, and gas bubbles cannot adhere to the fish skin. When a bubble is released onto the fish scale in water, it can maintain its spherical shape (Figure 25a) [46]. The bubble BCA is measured to be 155° ± 2.5°. The bubble will roll off immediately if the fish scale is tilted to 9° (Figure 25b). The result demonstrates that the fish scale shows underwater superaerophobicity, allowing fish skin to have tremendous anti-bubble ability. Such a bubble repellence prevents gas bubbles from attaching to the fish skin. Figure 25c–f reveals the reason why the surface of the fish scales is superaerophobic underwater. Fish scales exhibit hydrophilicity in the air because they are composed of hydrophilic chemical composition and hierarchal surface microstructures. Water entirely wets the surface microstructure of fish scales (Figure 25c). In water, the rough microstructures on a fish scale are filled with water (Figure 25d). A film of water likes being trapped by the surface microstructures. Water has a natural ability to repel incompatible gas. When a bubble comes in contact with the fish scale, it will be prevented from effectively contacting the rough microstructures of the fish scale by this trapped water layer (Figure 25e). The gas in the bubble can hardly replace the water in the rough surface microstructure because such a process is blocked by the trapped water. As a result, the underwater bubble maintains a spherical shape over time on the hydrophilic fish scale and just touches the peaks of the rough microstructures of the fish scale (Figure 25f). The contact between the bubble and the fish scale agrees well with the Cassie model in water (an underwater version) [36,314]. Since the surface microstructures do not allow underwater bubbles to effectively touch the scale surface, the fish scales show underwater superaerophobicity. In general, underwater superaerophobic materials have a great ability to repel gas in water.

**Figure 26:**
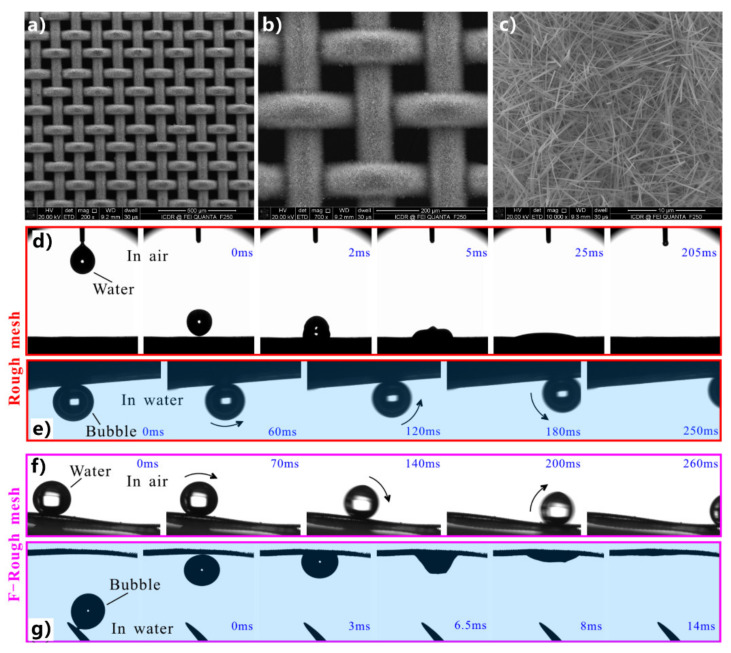
Underwater superaerophobicity and superaerophilicity of the nanoneedles-structured copper mesh. (**a**–**c**) Microstructure of the copper mesh with nanoneedles structure. (**d**) A water droplet falling onto the structure mesh in the air. (**e**) Underwater bubble rolling away on the structured mesh. (**f**) The rolling process of a water droplet on the fluoroalkylsilane-modified rough mesh in the air. (**g**) Releasing a bubble onto the modified rough mesh in water. Reprinted from ref. [327].

Inspired by fish scales, underwater superaerophobicity can be easily obtained by producing micro/nanostructures on a hydrophilic (high surface energy) substrate [315,316,317,318,319,320,321,322,323,324]. For example, Yong et al. produced hierarchical microstructures on an inherently hydrophilic silicon surface by a femtosecond laser [46]. The laser-ablated silicon surface is composed of periodic hierarchical micromountains with a size of 7–8 μm and a period of ~10 μm. Abundant nanoscale particles are also decorated on the surface of the micromountains. Water droplets can easily spread out on the as-prepared surface. The WCA is only 5° ± 1°. Therefore, the microstructures make the silicon surface change from a hydrophilic state to a superhydrophilic state. After immersion of the samples in water, a bubble on the untreated flat silicon surface has a BCA of 125° ± 2° and can adhere firmly to the surface of the silicon. In contrast, the bubble on the laser-structured silicon surface has a BCA of 162° ± 2°. The bubble can roll away easily with a bubble SA (BSA) of 2°. Therefore, after femtosecond laser processing, the silicon surface exhibits superaerophobicity and ultralow adhesion to bubbles underwater. Dorrer et al. prepared grass-like nanostructures with a high degree of roughness on the silicon surface by an anisotropic etching process [325]. The poly(*N*,*N*-dimethylacrylamide) (PDMAA) polymer was subsequently immobilized at the surface of the nanograss. If a bubble was released onto the surface of the structured silicon (from below) in water, the silicon kept the bubble spherical. The bubble easily rolled away as the sample was tilted at 5°. Therefore, the PDMAA-coated rough surface is underwater superaerophobic and has remarkable repellence to bubbles in the water. Xu et al. fabricated a hybrid film composed of GO and gold nanoparticles (GNPs) [326]. The natural hydrophilicity of the GO nanosheet and the hierarchical microstructure of the aggregated GNPs allow the water to easily wet and fill in the crevices of the hybrid film. The GO/GNP film is superaerophobic and exhibits very low adhesion to gas bubbles in a water medium. The hybrid films can be applied to flexibly manipulate bubbles in the water. The bubbles were successfully controlled to move by the homemade tools coated with the underwater superaerophobic hybrid film, without bubbles adhesion/residuals on the tool surface. The merging/reaction of different bubbles was also achieved. Yong et al. prepared Cu(OH)_2_ nanoneedles structure on the wire of a copper mesh by simply immersing the mesh into the solution of (NH_4_)_2_S_2_O_8_ and NaOH for 5 min [327]. The resultant mesh was uniformly coated with nanoneedles with a diameter of 150–260 nm and a length of 10–15 µm (Figure 26a–c). The structured mesh becomes superhydrophilic in the air (Figure 26d) and shows superaerophobicity in water. An underwater gas bubble on the rough mesh has a BCA of 161.5° ± 2.5°. Once the mesh is tilted to 5.5°, the bubble will start to roll off (Figure 26e). The structured copper mesh strongly repels the underwater bubbles, and the bubbles are difficult to stably stay on the superaerophobic surface. Sun et al. demonstrated that constructing underwater superaerophobic nano-porous architecture on electrode surface can promote the electro-catalytic performance of catalytic material during the gas evolution performance reaction [328]. The underwater superaerophobic electrode surface only provides a minimal contact area and extremely low adhesive force to the gas bubbles, so the generated gas bubbles naturally leave the solid electrode surface with ease. For example, nanostructured MoS_2_ film exhibited superaerophobicity in water. The adhesion of the generated gas bubbles on the MoS_2_ electrode surface was reduced by order of magnitude than the common electrode [328]. With the as-formed gas bubbles being timely removed, the working area did not shrink and maintained constant, resulting in very high efficiency of gas evolution.

Unlike the fish scales, the lotus leaf reflects the light like a silver mirror underwater because there is a trapped air film between the surface of the lotus leaf and the surrounding liquid environment. When a bubble comes in contact with the lotus leaf in water, it will break up immediately as long as it touches the leaf surface. The bubble spread out quickly along the leaf surface within a very short time (Figure 25g). Finally, the BCA of the bubble closes to 0°, like the bubble being entirely absorbed by the lotus leaf. Therefore, the surface of the lotus leaf shows underwater superaerophilicity and can absorb underwater gas [46]. Figure 25h–k depicts the formation mechanism of the superaerophilicity of the lotus leaf in water. Water on the superhydrophobic lotus leaf is in the Cassie state. Air is trapped in the surface microstructures of the lotus leaf, forming an air cushion between the lotus leaf and the water (Figure 25h). Even though the lotus leaf is immersed in water, the air film is also maintained between the lotus leaf and water because the superhydrophobic surface microstructure strongly repels water (Figure 25i). Water only touches the peaks of the rough microstructures of the lotus leaf. If a bubble further contacts the lotus leaf, the bubble will connect with the trapped air film over the lotus leaf. The high pressure of the bubble drives the gas in the bubble into this air film, and the bubble spreads out rapidly along with the broad air film (Figure 25j) [46,314]. As a result, the bubble merges into the trapped air layer (Figure 25k). If the superhydrophobic rough surface is wide enough, the surface can completely absorb the bubbles in the water. Therefore, the superhydrophobic lotus leaf surface is superaerophilic in water. The materials with underwater superaerophilicity have a remarkable ability to absorb and capture gas bubbles underwater.

Inspired by lotus leaves, underwater superaerophilicity can be easily obtained by producing micro/nanostructures on a hydrophobic (low-surface-energy) substrate [329,330,331,332,333,334,335,336,337,338]. For example, Yong et al. fabricated hierarchical micro-/nanostructures on a hydrophobic PDMS substrate by femtosecond laser ablation [46]. The laser-ablated area was covered with cauliflower-like structures with a size of several micrometers and fine nanoscale protrusions. The structured PDMS surface becomes superhydrophobic. When the resultant PDMS surface was submerged in water, and a bubble came in contact with the sample surface, the bubble spread out quickly on the structured surface within 35 ms. Finally, the bubble was entirely absorbed by the structured PDMS surface. The BCA is as low as ∼0° because the gas-water interface is very close to the sample surface, indicating that the structured PDMS surface exhibits underwater superaerophilicity. Dorrer et al. used Teflon-like perfluoroalkoxy (PFA) to modify the nanograss-structured silicon surface [325]. The resultant surface shows superhydrophobicity in air, with a WCA of 178 ± 1° and a WSA of <5°. As the surface was submerged in water, the air remained enclosed inside the surface structure. A bubble that was dispensed onto the surface could disappear into the surface structure. Therefore, the PFA-covered surface is superaerophilic in water. Xue et al. produced underwater superaerophilic microstructures on the surface of a copper cone via the dip-coating method [339]. The copper cone was obtained by gradient electrochemical etching. Then, the hydrophobic SiO_2_ nanoparticles were deposited on the copper cone. The water droplet remained spherical and could not stick to the resultant copper cone with a WCA of 160.5° ± 2.5°. Whereas a bubble has a BCA of ~0° on the copper surface in water, the structured copper cone was endowed with underwater superaerophilicity and gas-absorbing ability. Ma et al. fabricated underwater superaerophilic polyethylene (PE) sheet by sandpaper rubbing and coating with superhydrophobic nanosilica particles [340]. When the underwater superaerophilic sheet was cut into the triangles, the captured gas bubbles could be directionally and spontaneously transported by the trapezia sheet. The high driven force for bubble movement results from the superaerophilic shape-gradient morphology. Yong et al. used fluoroalkylsilane to modify the nanoneedles-structured copper mesh [327]. The mesh thus switched to superhydrophobic (Figure 26f). In water, a bubble that contacted the mesh spread quickly over the mesh (Figure 26g). The BCA of the bubble was as low as 6.5°, demonstrating the underwater superaerophilicity of the mesh. Lu et al. proposed a strategy to improve the oxygen-reduction-reaction performance (i.e., accelerating the gas-diffusion process and electron transport) through designing superaerophilic micro-/nanostructures on the electrode [341]. The cobalt-incorporated nitrogen-doped carbon nanotubes were directly grown on carbon-fiber paper. The porous nanotubes were also modified with PTFE. The as-prepared catalyst has a stable superaerophilic property in both acidic and alkaline liquids. A stable oxygen-gas layer formed on the catalyst surface in the electrolyte solution, which remarkably accelerated the gas-diffusion process during the oxygen reduction reaction.

Usually, most superhydrophilic microstructures show superaerophobicity, and most superhydrophobic microstructures show superaerophilicity in a water medium. Therefore, underwater superaerophobicity can be obtained by creating microstructures on a hydrophilic substrate, while underwater superaerophilicity can be obtained by creating microstructures on a hydrophobic substrate. Bubbles have opposite wetting behavior on the underwater superaerophobic and superaerophilic surfaces in water, respectively [303,304]. The underwater superaerophobic surfaces can repel bubbles so that bubbles are difficult to adhere to the material surface in water. In contrast, bubbles can easily spread over underwater superaerophilic surfaces and be absorbed by solid surfaces. The unique bubble wettability enables the underwater superaerophobic and superaerophilic materials to become an advanced tool for manipulating underwater bubbles.

### 6.2. Selective Passage of the Bubbles

**Figure 27:**
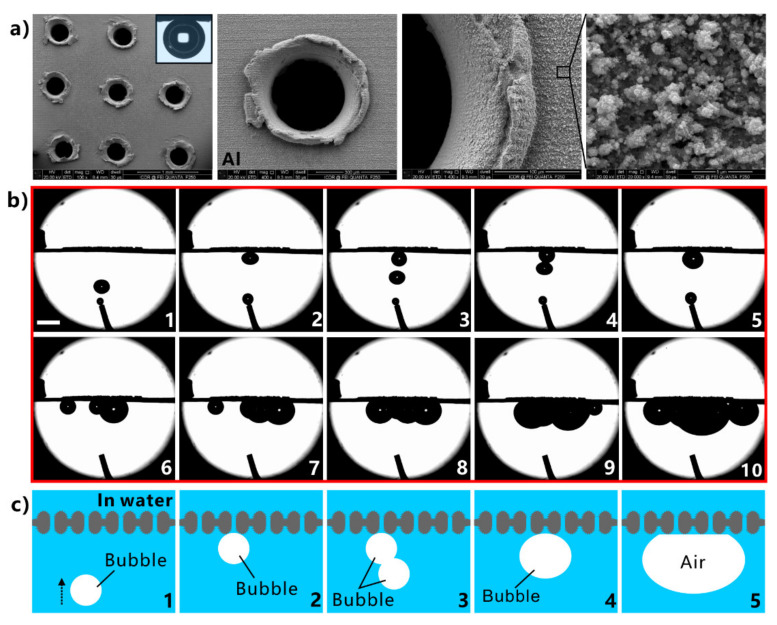
Underwater interception effect of the superaerophobic porous sheet to bubbles. (**a**) SEM images of the porous aluminum sheet. Inset shows a bubble on the structured aluminum substrate in water. (**b**) Bubbles continuously rising to the superaerophobic porous sheet underwater. (**c**) Schematic mechanism of the interception function of the underwater superaerophobic porous sheet to underwater bubbles. Reprinted with permission from ref. [46]. Copyright 2017, American Chemical Society.

The influence of the underwater gas wettability on the dynamic behavior of bubbles through a porous sheet in water was investigated by Yong and co-authors [46,342,343,344]. They fabricated an underwater superaerophobic porous sheet, as shown in Figure 27a [46]. Many holes with a diameter of ~312 μm were prepared on a thin aluminum foil by a mechanical drilling process, which across through the sheet. Then, hierarchical micro-/nanostructures were induced on both sides of the foil via femtosecond laser processing (Figure 27a). The rough, porous aluminum sheet exhibits underwater superaerophobicity. In water, tiny bubbles on such a sheet have a BCA of 151° (inset of Figure 27a). Figure 27b shows that air bubbles were continuously released below the underwater superaerophobic porous sheet in the water medium. As the first bubble rose to the sheet, it was intercepted by the porous sheet and stopped from rising. Likewise, the subsequent bubble was also stopped by this sheet. Once these bubbles touched each other, they would merge into a bigger bubble. No matter how many bubbles rose to the sheet, the underwater superaerophobic sheet did not allow them to pass through. The result indicates that the underwater superaerophobic porous sheet has the interception function to the bubbles in a liquid, which does not allow gas bubbles to pass through.

The bubble-interception effect of the underwater superaerophobic porous sheet is ascribed to the underwater gas repellence of the surface microstructure of the porous sheet, as shown in Figure 27c. In liquid, the surface microstructure and the pores of the sheet are completely wetted by water. The underwater superaerophobicity of the surface microstructure caused by the trapped water film repels all bubbles touching the surface of the sheet. The water that fills the pores also blocks the passage of bubbles through the pores. So, no matter how many bubbles rise to the underwater superaerophobic porous sheet, they are intercepted and stopped below the sheet.

**Figure 28:**
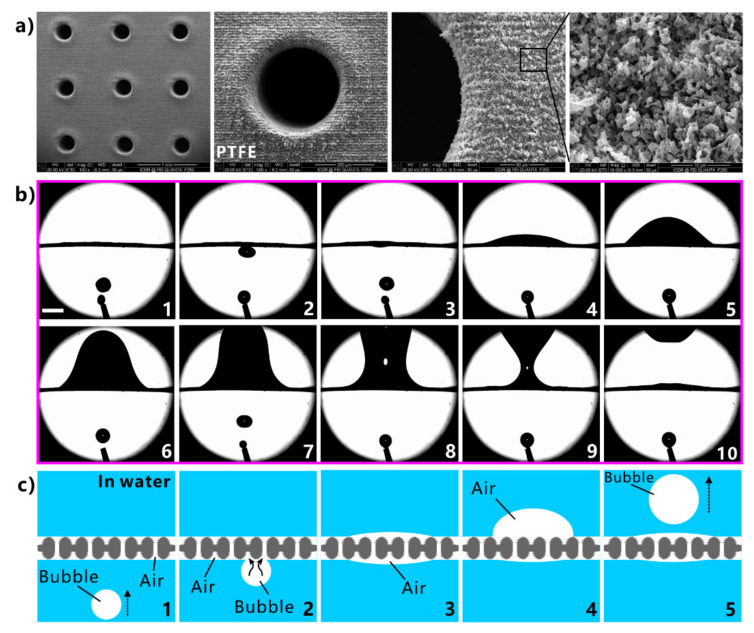
Ability of bubbles passing through an underwater superaerophilic porous sheet in water. (**a**) Morphology of the superaerophilic porous PTFE sheet. (**b**) Process of continuously releasing bubbles below the porous sheet underwater. (**c**) Schematic mechanism of the passage of the bubbles through the underwater superaerophilic porous sheet in water. Reprinted with permission from ref. [46]. Copyright 2017, American Chemical Society.

The underwater superaerophilic porous sheet shows a very different function to the behavior of bubbles in water compared to the underwater superaerophobic porous sheet. A porous sheet with underwater superaerophilic surface microstructures was obtained by producing open microholes (diameter of the holes = ~287 μm) and rough surface microstructures on an intrinsically hydrophobic thin PTFE sheet (Figure 28a) [46]. The as-prepared porous sheet is superaerophilic in water with a BCA of ~0° to a small air bubble. The experiment of continuously releasing bubbles below this sheet was also performed underwater, as shown in Figure 28b. As the first bubble came in contact with the porous sheet, it was instantaneously absorbed by the sheet due to the superaerophilicity of the sheet in water. Similarly, subsequent bubbles were also absorbed by the lower side of the sheet. After a certain accumulation of bubbles, the absorbed gas in the sheet gradually bulged on the upper side of the sheet. A convex air bulge appeared. The gas volume of the air bulge continuously increased, resulting in the shape of the air bulge gradually growing. When the buoyancy force of the air bulge was sufficient to overcome the adhesion between the bulge and the upper surface of the sheet after gas accumulation, the air bulge finally detached the porous sheet and rose as a new bubble, one after another. With the repetition of the abovementioned process, all bubbles successfully passed through the underwater superaerophilic porous sheet.

The bubble-passage process through the porous sheet is schematically shown in Figure 28c. The underwater superaerophilicity and porous structure play a critical role in allowing bubbles to permeate through the porous sheet. The underwater superaerophilic surface is generally superhydrophobic. When the underwater superaerophilic porous sheet is put in water, air films will be trapped on the surface microstructures of both sides of the sheet (Step 1). The pores of the sheet are filled with air, which connects the two air films on either side of the sheet. When a bubble rises and touches the lower side of the sheet (Step 2), the gas in the bubble will flow into the trapped air film (Step 3). As a result, the bubble merged into the air film around the sheet because of the superaerophilicity of the sheet surface. The volume of the trapped gas around the sheet increases with continuously absorbing bubbles. As the pressure of the trapped gas gradually increases, the gas lifts the water on the upper side of the sheet at some point, resulting in a gas bulge forming above the porous sheet (Step 3). The volume of the air bulge gradually increases (Step 4). Finally, the air bulge detaches the top surface of the porous sheet and rises as a new bubble when the buoyancy of the bulge is large enough (Step 5). Overall, all the bubbles successfully across the underwater superaerophilic porous sheet.

It is demonstrated that the underwater superaerophobic porous membrane can intercept bubbles, while the underwater superaerophilic porous membrane allows bubbles to pass through in water [46,342,343,344]. These two kinds of superwetting materials show different effects on bubbles. The particular gas wettability offers a new idea to separate liquid and gas. The tiny gas bubbles can be collected or removed from the liquid using underwater superaerophobic and underwater superaerophilic porous materials.

### 6.3. Collection of Gas Bubbles in Liquid

**Figure 29:**
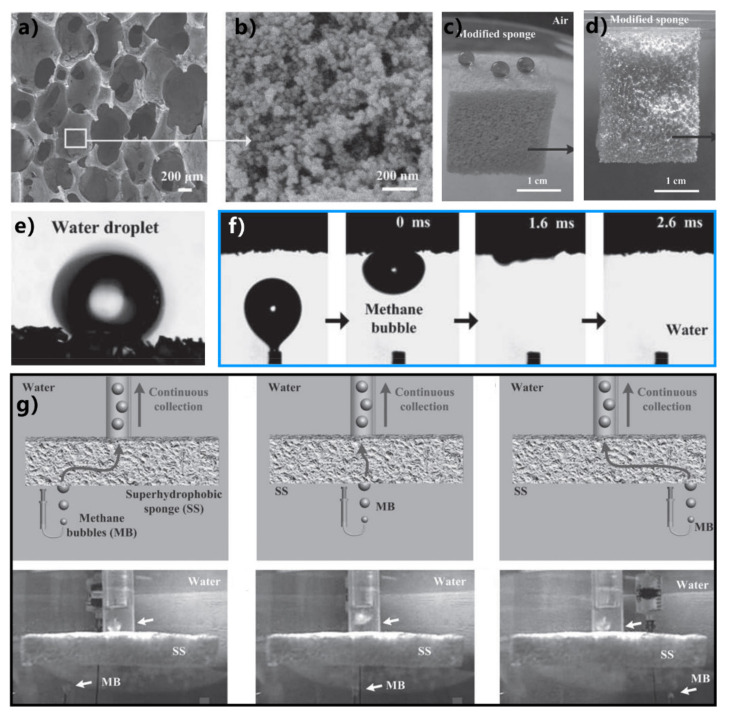
Collecting bubbles by a superaerophilic sponge underwater. (**a**,**b**) Surface microstructure of the PU sponge coated with hydrophobic-nanoparticle/polymer composite. (**c**) Water droplets on the modified sponge. (**d**) The modified sponge in water. (**e**) Water droplet rolling on the modified sponge. (**f**) Process of a bubble of methane contacting the sponge surface in water. (**g**) Collection of methane bubbles by using the modified sponge. Reprinted with permission from ref. [345]. Copyright 2012, WILEY-VCH Verlag GmbH & Co. KgaA.

In water, gas bubbles can freely spread on a superhydrophobic lotus leaf, but the absorption process is limited because the surface area of the lotus leaf is too small. Chen et al. proposed a method to absorb marine methane bubbles using superhydrophobic and underwater superaerophilic materials with high specific surface area [345]. The sponge with a rich porous structure was dipped in a sticky hydrophobic-nanoparticle/polymer composite solution and pulled up. After the evaporation of the organic solvent, the aggregations of the hydrophobic fumed silica nanoparticles cover the whole surface of the porous skeletons (Figure 29a,b). The composite coatings of low-free-energy nanoparticles change the wettability of sponges from hydrophilic to superhydrophobic, with a WCA of 157° (Figure 29c) and a WSA of <6° (Figure 29e). When the sponge was dipped into water, it was surrounded by an air film (Figure 29d). The wettability of the underwater bubble on the sponge was investigated by immersing the sponge in artificial seawater and allowing a bubble of pure methane to rise and contact the sponge surface. On the modified sponge, the coalescence of the underwater bubble and the gas film around the sponge occurred at a tremendous speed (within 3 ms) (Figure 29f). The bubble completely spread over the sponge surface and ultimately entered into the inner cavity of the sponge. Therefore, the modified sponge shows superaerophilicity in water and can absorb methane bubbles. A rigid plastic pipe was fixed onto the top of the underwater superaerophilic sponge to guide the absorbed methane bubbles through the pipeline (Figure 29g). When methane bubbles were continuously released below the sponge, they would rise and be absorbed by the sponge. Even though the sponge reached the saturated state with gas, it could continue to absorb bubbles as the extra methane gas was transported along the pipe (water was injected into the rigid pipe to observe bubble transport), allowing continuous bubbles absorption. Interestingly, the methane bubbles could be absorbed any part (from either the leaf part, the middle part, or the right side) of the underwater superaerophilic sponge and be transported along the pipe because of the natural porous network structure inside the sponge, as shown in Figure 29g.

Li et al. also achieved similar collection and transport of underwater methane bubbles via a cellulose-based aerogel [346]. As a kind of ultralight porous material, cellulose aerogel composed of the cellulosic polymer is characterized by rich porosity, low density, and large surface area. In their experiment, the cellulose aerogels were fabricated through a freezing-drying process, followed by methyltrimethoxysilane modification. The as-prepared cellulose aerogel exhibits strong hydrophobicity and underwater superaerophilicity. The aerogel showed suitable water repellence and could float effortlessly on the water. Once methane bubbles came in contact with the aerogel surface in water, they burst and were quickly absorbed into the aerogel. The underwater superaerophilicity endows the cellulose aerogel with excellent ability to capture methane bubbles. By integrating a pipe for transporting the collected gas away, the modified cellulose aerogel can continuously collect useful methane bubbles in a liquid.

**Figure 30:**
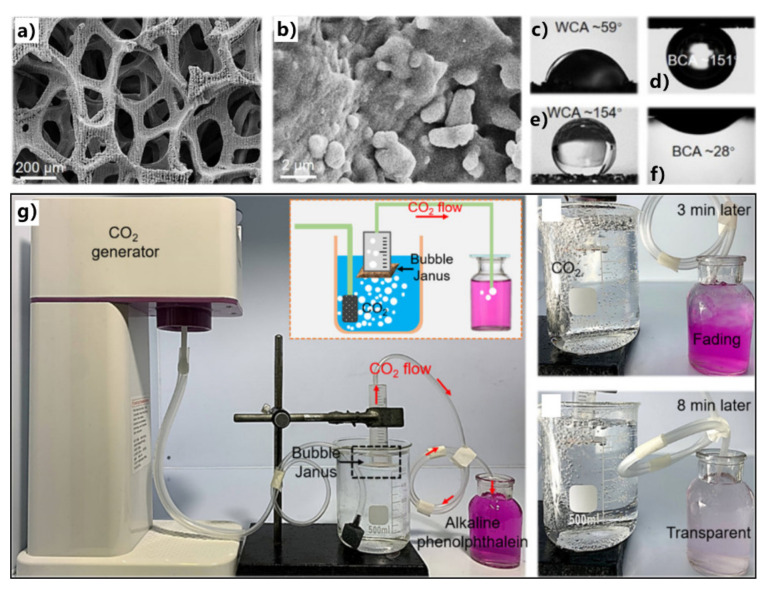
Collection of bubbles by a bubble Janus. (**a**,**b**) Microstructure of the laser-treated side of the copper foam. (**c**) Water droplet (in the air) and (**d**) underwater bubble on the laser-treated side of the foam. (**e**) Water droplet (in the air) and (**f**) underwater bubble on the untreated side of the foam. (**g**) Underwater carbon-capturing device based on the unidirectional bubble-transportation property of the Janus foam. Reprinted with permission from ref. [347]. Copyright 2020, American Chemical Society.

Zhu et al. fabricated a bubble Janus based on the copper foam (thickness ∼1 mm) [347]. The copper foam was firstly treated with a chemical coating to exhibit superhydrophobicity. Silica nanoparticles thus coated the smooth surface of the copper branches of the fresh foam. Then, one side of the copper foam was further ablated by an ultrafast laser. The micro/nanoscale composite structures (hollow porous microstructures, micro/nano-mastoids) were generated on the surface of each branch (Figure 30a,b). The laser-treated side became hydrophilic with a WCA of 59° to water droplet in the air (Figure 30c) and superaerophobic with a BCA of 151° to bubbles in water (Figure 30d). The untreated side maintained superhydrophobicity with a WCA of 154° to water droplets in the air (Figure 30e) and showed aerophilicity with a BCA of 28° to bubbles in water (Figure 30f). It was found that the as-prepared Janus foam just allowed underwater bubbles to pass from the laser-ablated side to the unablated side, while the opposite-direction passage was impracticable. Based on the unidirectional bubble-transportation property of the Janus foam, a device for underwater carbon capture was designed, as shown in Figure 30g. The Janus foam allowed carbon dioxide bubbles to penetrate through and enter the collecting chamber. As the captured gas was continuously injected into the alkaline phenolphthalein solution, the decolorization of phenolphthalein occurred, demonstrating that the carbon dioxide bubbles were successfully collected.

**Figure 31:**
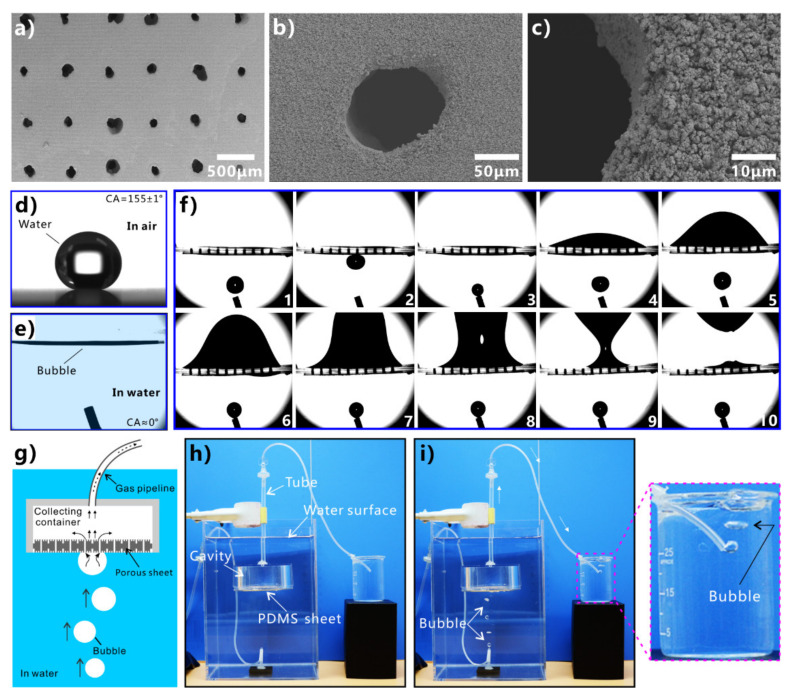
Underwater bubble-collection device composed of superhydrophobic and underwater superaerophilic porous sheet. (**a**–**c**) Morphology of the microholes and rough surface microstructures on the PDMS sheet. (**d**) A small drop of water on the structured sheet in the air. (**e**) Underwater air bubble on the structured sheet. (**f**) Passage of bubbles through the porous superwetting sheet in water. (**g**) Schematic of the designed collection device. (**h**) Photo of the artificial bubble-collection device. (**i**) Collection of bubbles from water. Reprinted with permission from ref. [342]. Copyright 2018, The Royal Society of Chemistry.

Superhydrophobic materials have a remarkable ability to repel water, and bubbles can pass through the underwater superaerophilic porous sheets in a liquid environment. Yong et al. designed an underwater bubble-collection device whose core component was an underwater superaerophilic porous sheet [342]. Microholes and rough surface microstructures were prepared on a PDMS sheet via mechanical drilling and femtosecond laser ablation, respectively (Figure 31a–c). The structured porous sheet shows both in-air superhydrophobicity (Figure 31d) and underwater superaerophilicity (Figure 31e). In water, bubbles can easily pass through the porous superwetting sheet (Figure 31f). As shown in Figure 31g,h, the backbone of the collection device is a cavity whose bottom is coated with the as-prepared underwater superaerophilic porous sheet. A thin tube is used to transport the collected gas, whose inlet end is connected to the enclosed cavity. In liquid, as the air bubbles below this device continually rose and arrived at the device one by one, all the bubbles were wholly absorbed by the bottom of the designed collection device; that is, all of the bubbles entered into the inner space of the device (Figure 31i). It was found that gas kept blowing out of the pipe, demonstrating that the underwater gas bubbles were successfully collected and transported along the pipeline (inset of Figure 31i). The superwettability of the as-prepared porous sheet plays a critical role in underwater gas collection. The sheet allows bubbles to penetrate it and be collected by the device because of its underwater superaerophilicity. Still, it simultaneously blocks water from entering into the collection device because of its superhydrophobicity. The gas species do not influence such a bubble-collection process, so various gas bubbles can potentially be collected.

### 6.4. Removal of Bubbles from Water

**Figure 32:**
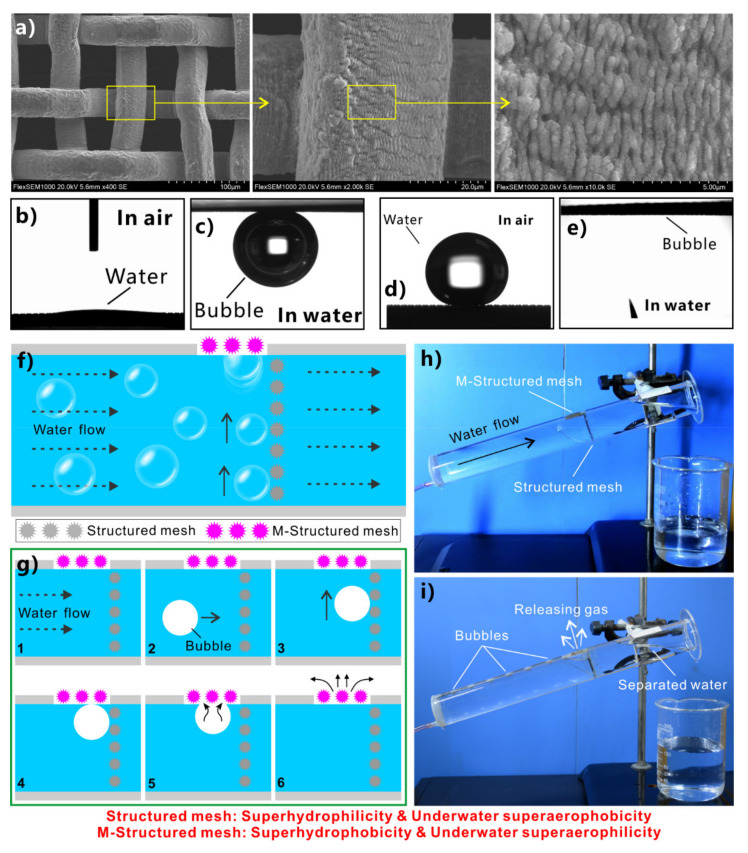
Removing bubbles from water flow by the combination of underwater superaerophobic and underwater superaerophilic meshes. (**a**) Surface microstructure of the laser-ablated stainless-steel mesh. (**b**) Water droplet (in the air) and (**c**) underwater bubble on the structured mesh. (**d**) Water droplet (in the air) and (**e**) underwater bubble on the M-structured mesh. (**f**) Schematic of a device composed of underwater superaerophobic and superaerophilic meshes for removing bubbles from the water in a pipeline. (**g**) The mechanism of the bubble-removing process. (**h**,**i**) Experimental result of removing bubbles in water flow by the artificial separation device: (**h**) before the appearance of bubbles and (**i**) after the appearance of bubbles. Reprinted with permission from ref. [348]. Copyright 2021, The Royal Society of Chemistry.

In some cases, bubbles are useless and harmful. The collection of these bubbles does not make any sense. The bubbles can be directly removed from the liquid. Yong et al. proposed a strategy for removing tiny bubbles from water flow in a pipeline [348]. The surface of a stainless-steel mesh was processed by a femtosecond laser. Periodic nanoscale ripple-like structures were generated on the wire surface (Figure 32a). The structured mesh exhibited superhydrophilicity in the air (Figure 32b) and underwater superaerophobicity (Figure 32c). The BCA of a bubble on the structured mesh is 153.6° ± 0.6°, and the BSA of the bubble is only 2.0° ± 2.0° in water. Once the mesh was further treated by low-surface-energy modification, the structured mesh (defined as the “M-structured mesh”) became superhydrophobic with a WCA of 152.5° ± 3.0° to water droplets in the air (Figure 32d). In water, a bubble could be quickly absorbed by the mesh, with a BCA of ~0° (Figure 32e). The result indicates that the M-structured mesh shows underwater superaerophilicity. As a result, two kinds of meshes with different wettabilities were fabricated. The structured mesh is superhydrophilic and underwater superaerophobic, while the M-structured mesh is superhydrophobic and underwater superaerophilic. Figure 32f depicts the schematic of a device for removing tiny bubbles from the water flow. The structured underwater superaerophobic mesh is perpendicularly inserted into the water pipe. There is a small hole in the wall of the pipe, which is located just in the front of the structured mesh and is covered with the M-structured underwater superaerophilic mesh. The working mechanism of water/gas separation is shown in Figure 32g. Water can wet and flow through the structured mesh freely because of the superhydrophilicity of the inserted mesh (Step 1). As the bubbles in water move forward with water flow and reach the structured metal mesh (Step 2), the remarkable bubble repellence of the underwater superaerophobic mesh enables the mesh to intercept bubbles so that all the bubbles stop forward (Step 3). The buoyancy will drive the bubbles to rise until they touch the M-structured mesh (Step 4) and are absorbed by such an underwater superaerophilic mesh (Step 5). The gas in the bubbles can quickly pass through the M-structured mesh and be released into the external environment (Step 6). On the other hand, the water in the pipeline is prevented from flowing out of the pipe because the M-structured mesh also has excellent superhydrophobicity and water repellence. The removal capacity of bubbles in water flow was also successfully verified through experiment, as shown in Figure 32h,i. The inserted structured mesh intercepted all the bubbles in the water flow. Finally, these bubbles were expelled from the pipeline through the M-structured mesh on the pipe wall. There were no bubbles in the separated water, demonstrating that all the bubbles in the water flow were completely removed.

Cu(OH)_2_ nanoneedles can grow on the surface of a copper mesh by a one-step chemical reaction. The nanoneedles-structured copper mesh is superhydrophilic and superaerophobic underwater. The wettability of the rough mesh can switch to superhydrophobicity and underwater superaerophilicity after fluoroalkylsilane modification. Yong et al. also successfully removed bubbles from a water flow by combining these two kinds of copper meshes, the same as the abovementioned strategy [327]. The superhydrophilic and underwater superaerophobic copper mesh was used to intercept the gas bubbles because the underwater superaerophobicity prevented bubbles from penetrating the mesh. The intercepted bubbles were released out of the water pipe (or microchannel) through a superhydrophobic and underwater superaerophilic copper mesh. Therefore, gas bubbles were easily removed from the water flow.

Such effective removal of bubbles in liquid is achieved by combining underwater superaerophobic and superaerophilic porous membranes, which have different bubble wettabilities. The separation strategy can avoid the harm caused by bubbles in microfluidic systems, liquid-detecting instruments, infusion tubes, etc.

Whether it is collection or removal, the function of separating gas from a liquid is successfully realized by the superaerophobic or superaerophilic porous materials. Both bubble removal and collection processes are based on the gas self-transportation from the water side to the air side through the superwetting porous sheet [349]. The removal or collection of underwater bubbles achieves the purpose of the separation of liquid and gas. Liquid/gas separation is an essential technology in chemical engineering, energy, and the environment.

## 7. Conclusions and Future Perspectives

This review systematically summarizes the emerging applications of surface superwettability in solid/liquid/gas separation. Much attention is focused on generalizing the well-developed superwettability-based oil/water separation process to treat other types of liquid/liquid or liquid/gas mixtures. The connotation of liquid repellence in separating liquids from solid materials is pointed out for the first time. The liquid-repellent materials (such as superhydrophobic surfaces and superoleophobic surfaces) cannot be wetted by liquids, keeping the liquids away from the solid materials. The superhydrophobic and superoleophilic porous membranes allow oil to pass through them, but the membranes simultaneously intercept water. By contrast, water can permeate through the prewetted underwater superoleophobic porous membranes, but oil cannot. The oil can also be directly absorbed and collected from water by using superhydrophobic 3D oil-absorption materials. Therefore, the mixtures of oil and water are separated by the superwetting porous materials. Inspired by the process of oil/water separation, the separation of the mixture of liquid polymers and water can also be achieved by the underwater superpolymphobic porous membranes, which allow water to permeate through but intercept liquid polymers. With the development of underwater superaerophobic and superaerophilic materials, these materials are also used to achieve liquid/gas separation because of the selective passage effect of the bubbles through the underwater porous superaerophobic/superaerophilic membranes. Bubbles in liquids can be collected or removed by using underwater superaerophobic and superaerophilic porous materials.

Although the materials with superwettability have shown great success and advantages in separation applications, the superwettability-based separation technology still has great development space in the future. Firstly, the current separation is aimed at a certain amount of liquid and gas. The separation of a very tiny amount of liquid and gas or even molecular level remains further investigated. Secondly, the separation technology must eventually be able to meet the needs of large-scale applications. The cost, simplicity, repeatability, and durability of the separation process should be considered and optimized. Thirdly, in most reported separation cases, single pure water, oil, and gas were separated. However, solids, liquids, and gases are often complex and made up of many components in the real world. It is still a challenge to separate complex mixtures that consist of more than two chemicals. Last but not least, the current separations based on surface wettability are still in the laboratory stage. The separation methods and materials should be marketed to solve practical separation problems.

The wettability-based sold/liquid/gas separation has many advantages over traditional separation materials and methods. We believe that the superwettability-based separation has great potential applications in chemical manufacturing, wastewater treatments, environmental protection, energy use, oil production and separation, mineral industry, biological cell incubations, health care, agricultural breeding, miniature reactor technologies, microfluidics, boiling processes, and so on.
